# Design space determination to optimize DNA complexation and full capsid formation in transient rAAV manufacturing

**DOI:** 10.1002/bit.28508

**Published:** 2023-07-21

**Authors:** Qiang Fu, Yong Suk Lee, Erica A. Green, Yongdan Wang, So Young Park, Ashli Polanco, Kelvin H. Lee, Michael Betenbaugh, David McNally, Seongkyu Yoon

**Affiliations:** 1 Department of Biomedical Engineering and Biotechnology, University of Massachusetts Lowell, Lowell, Massachusetts, USA; 2 Department of Pharmaceutical Sciences, University of Massachusetts Lowell, Lowell, Massachusetts, USA; 3 Department of Chemical and Biomolecular Engineering, University of Delaware, Newark, Delaware, USA; 4 Department of Chemical Engineering, University of Massachusetts Lowell, Lowell, Massachusetts, USA; 5 Department of Chemical and Biomolecular Engineering, Johns Hopkins University, Baltimore, Maryland, USA; 6 MassBiologics, University of Massachusetts Chan Medical School, Mattapan, Massachusetts, USA

**Keywords:** Design of Experiments (DoE), Design Space (DS), Gene therapy manufacturing, Process Parameters (PPs), Product Quality Attributes (PQAs), Recombinant adeno-associated virus (rAAV)

## Abstract

Recombinant adeno-associated virus (rAAV) vectors are a promising platform for in vivo gene therapies. However, cost-effective, well-characterized processes necessary to manufacture rAAV therapeutics are challenging to develop without an understanding of how process parameters (PPs) affect rAAV product quality attributes (PQAs). In this work, a central composite orthogonal experimental design was employed to examine the influence of four PPs for transient transfection complex formation (polyethylenimine:DNA [PEI:DNA] ratio, total DNA/cell, cocktail volume, and incubation time) on three rAAV PQAs related to capsid content (vector genome titer, vector genome:capsid particle ratio, and two-dimensional vector genome titer ratio). A regression model was established for each PQA using partial least squares, and a design space (DS) was defined in which Monte Carlo simulations predicted < 1% probability of failure (POF) to meet predetermined PQA specifications. Of the three PQAs, viral genome titer was most strongly correlated with changes in complexation PPs. The DS and acceptable PP ranges were largest when incubation time and cocktail volume were kept at mid-high setpoints, and PEI:DNA ratio and total DNA/cell were at low-mid setpoints. Verification experiments confirmed model predictive capability, and this work establishes a framework for studying other rAAV PPs and their relationship to PQAs.

## 1 ∣ INTRODUCTION

Adeno-associated virus (AAV) has become a popular platform vector for in vivo gene therapy due to its nonpathogenicity, low immunogenicity, and wide serotype-dependent tissue tropism compared to other viral vectors (Athanasopoulos et al., 2000; Ferrari et al., 1997; Patel et al., 2019). Multiple recombinant AAV (rAAV) gene therapies have been approved by regulatory agencies, and many other therapies are in clinical trials to treat conditions ranging from blood disorders to neuromuscular diseases (Au et al., 2021). Although rAAV therapies show great promise in the clinic, there are many challenges surrounding rAAV production that limit the ability of manufacturers to produce sufficient vector quantities for clinical and commercial use (Fu et al., 2023; Wang et al., 2023).

The manufacturing process for a therapeutic can be developed based on the quality by design (QbD) framework described in the International Council for Harmonization of Technical Requirements for Pharmaceuticals for Human Use (ICH) Q8(R2) guideline (Holm et al., 2017; ICH Expert Working Group, 2009; Rathore & Winkle, 2009; Yu et al., 2014; Yu, 2008). The QbD process starts with the identification of a quality target product profile (QTPP) based on characteristics important for dosing and administering a therapeutic. From there, physical, chemical, or biological properties that must be within a defined range to guarantee the QTPP, known as critical quality attributes (CQAs), are defined from all possible product quality attributes (PQAs). Critical process parameters whose operating ranges directly contribute to keeping CQAs within specifications are then identified from all PPs following risk assessments and design of experiments (DoE)-based studies that explore the design space (DS) for PP settings. Exploring the relationship between PQAs and PPs, especially in the early development of rAAV biomanufacturing, is worthwhile because CQAs are still unidentified and the approach to rAAV characterization is not yet mature. The nuanced process of transient transfection further complicates this relationship. Furthermore, risk assessment and DS exploration based on QbD principles have been applied during the development of many monoclonal antibody therapies (Bhatia et al., 2016; Böhl et al., 2021; CMC Biotech Working Group, 2009), but they have only recently been implemented for rAAV gene therapy products (Committee for Advanced Therapies [CAT], 2020; Spark Therapeutics, 2017). As a result, the current body of literature exploring rAAV process DSs, which PQAs and PPs are critical, and the associated relationships between PQAs and PPs are limited (Meade et al., 2021; Tanaka et al., 2020; Tustian & Bak, 2021). Results from studies to identify target operating ranges for PPs and test how robust PQAs are against fluctuations in PP setpoints would aid in the design of process control and product quality monitoring strategies for manufacturing safe and effective rAAV.

Recent publications have used DoE-based studies to optimize triple-plasmid transfection of Human Embryonic Kidney 293 (HEK293) cells, which is currently the most commonly used rAAV manufacturing process (Robert et al., 2017; Srivastava et al., 2021). These studies have explored how rAAV vector genome titer trends with PPs for process steps related to (1) the complexation reaction between the transfection reagent and plasmid DNA and (2) HEK293 cell culture (Grieger et al., 2016; Gu et al., 2018; Guan et al., 2022; Zhao et al., 2020), but do not investigate how these PPs related to other PQAs While viral genome (VG) titer is the dose-determining quality attribute for rAAV, it is only one of many PQAs related to rAAV safety and efficacy that are important to optimize when setting PP ranges to develop a robust production process (Green & Lee, 2021; Kizhedath & Glassey, 2017; Wright, 2021). Other PQAs, such as vector potency, product-related impurity levels, and process-related impurity burden (residual host cell protein and DNA etc.), should also be monitored to ensure the efficacy and safety of rAAV drug products (Green & Lee, 2021; Kizhedath & Glassey, 2017; Wright, 2021).

There is particular interest in optimizing rAAV manufacturing processes to limit the generation of product-related impurities (Fu et al., 2019; Li et al., 2020). These undesired vector forms include capsids that are empty, contain a truncated (partial) therapeutic genome cassette, and contain incorrectly packaged (misencapsidated) host cell or plasmid DNA fragments (Green & Lee, 2021; Wright, 2021). Although the impacts of these vector populations on patient outcomes are not fully understood (Wang et al., 2019; Wright, 2014), they do not provide a direct therapeutic benefit and increase impurity loads for downstream processing (Dickerson et al., 2021; Fu et al., 2019; Werle et al., 2021). The relative amounts of full, partial/misencapsidated, and empty vectors can be monitored by analytical ultracentrifugation (AUC) or transmission electron microscopy (TEM) for quality release testing. However, the low throughput and inability to resolve specific partial/misencapsidated vector populations by AUC or TEM significantly hinder process optimization efforts (Green & Lee, 2021; Wright, 2021).

Development of assays to monitor specific vector populations in crude samples during upstream process development poses a challenge due to the low concentration of rAAV relative to impurities (Ai et al., 2017; Mayginnes et al., 2006; Werling et al., 2015). Higher throughput monitoring of full, partial/misencapsidated, and empty vectors could be achieved by combining quantitative polymerase chain reaction (qPCR) or digital PCR (dPCR) with enzyme-linked immunosorbent assay (ELISA) to quantify encapsidated DNA sequence abundance and capsid particle (CP) titers, respectively and this data can be used to refine PP ranges. VG titers measured using single-target qPCR or dPCR are generally used to quantify full capsids (Aurnhammer et al., 2012; Martinez-Fernandez de la Camara et al., 2021). The ratio of full to total capsids can be estimated by calculating the ratio of genome to capsid titers using single-target qPCR or dPCR VG and ELISA CP measurements (Kuck et al., 2007; Lock et al., 2010; Wu et al., 2022). It has also been shown that the use of a multi-dimensional PCR assay to measure rAAV genome titer can provide insight into what percentage of amplicon-containing capsids may have full versus partial genomes. Two-plex digital droplet PCR (ddPCR) assays targeting combinations of the promoter, transgene, and polyadenylation (polyA) tail have shown that only 50–70% of single-stranded DNA containing capsids in rAAV samples contain double positive genomes, while up to 96% of plasmid DNA controls contained both targets, suggesting that multi-amplicon PCR analysis can discern full from partial capsids (Furuta-Hanawa et al., 2019; Hayes & Dobnik, 2022; Zanker et al., 2022).

Analysis of how VG- and CP-related quality attributes trend between conditions in DoE studies can be used to set PP ranges to optimize PQAs. In this work, we studied how PP operating ranges for the polyethyleneimine (PEI) and plasmid DNA complexation step of a suspension cell based, transient transfection upstream rAAV bioprocess and relate rAAV PQAs beyond VG titer. Four complexation PPs, PEI:DNA ratio, total DNA mass per cell, transfection cocktail volume, and incubation time, were identified as important to study based on literature (Grieger et al., 2016; Gu et al., 2018; Guan et al., 2022; Zhao et al., 2020). Complexation PP ranges explored and fixed PPs related to cell culture and gene expression, the cell density at transfection and plasmid ratio were set based on literature reviews (Grieger et al., 2016; Gu et al., 2018). A central composite orthogonal (CCO) DoE was used to relate the four input factors (PPs) to three output responses (PQAs): VG titer, vector genome to capsid particle (VG:CP) ratio, and two-dimensional (2D) VG titer ratio between the polyA tail and enhanced green fluorescent protein (eGFP) transgene. Central Composite Circumscribed (CCC) design was employed because the edge points of all factors were known based on physical and process limits, and additional levels for each factor can be explored compared to other Response Surface Methodology (RSM) designs. CCC Orthogonal (CCO) design (one type of CCC) was used so runs could be performed in three batches each containing three center points for orthogonal verification. VG titer and 2D VG titer ratio were calculated with qPCR data, and VG:CP ratio was calculated with qPCR and ELISA data. Using these data, a regression model for each PQA was established in MODDE^®^ using partial least squares (PLS). These models showed that of the three studied PQAs, VG titer is most heavily influenced by changes in complexation parameters. A DS of acceptable PP operating ranges was then established that met specifications for VG titer, VG:CP ratio, and 2D VG titer ratio that were set during experimental design. Verification experiments performed with PP setpoints inside and outside of the DS verified the probable < 1% failure of conditions within the DS to meet the PQA specifications, and this low probability of failure (POF) region is largest when cocktail volume and complexation time were at their mid or high setpoints and PEI:DNA ratio and total DNA/cell were at their low-mid setpoints.

## 2 ∣ MATERIALS AND METHODS

### 2.1 ∣ Experimental design

Experimental design was performed in MODDE^®^ (V13.0, Sartorius, Sweden). Four transfection input PPs, were studied using a CCO DoE. Low/high PP setpoints were selected based on previous literature reports and physical constraints of the transfection system (Table 1) (Chahal et al., 2014; Grieger et al., 2016; Gu et al., 2018; Meade et al., 2021; Zhao et al., 2020). The CCO DoE enabled study of 33 shake flask conditions with PPs at varying setpoints, nine of which were replicated center points (Supporting Information: Table S1).

### 2.2 ∣ Plasmid preparation

*Escherichia coli* stabs containing the plasmids needed for triple transient transfection were purchased from Addgene. The specific plasmids used were pAdDeltaF6 (Addgene 112867), pAAV2/2 (Addgene 104963), and pAAV-CMV-GFP (Addgene 67634). The *E. coli* were grown in Luria-Bertani broth supplemented with 100 μg/mL ampicillin before plasmid preparation. Plasmids were extracted and purified with the ZymoPURE II Plasmid Maxiprep Kit (Zymo Research) and then sterilized via 0.22 μm filtration. Plasmid concentration was measured by A260 on a Nanodrop^™^ OneC (Thermo Fisher). The A230/260 and A260/280 ratios measured on the Nanodrop were used to confirm plasmid preps were of sufficient quality.

### 2.3 ∣ Mammalian cell culture

HEK293 cells (Mass Biologics) adapted to BalanCD HEK293 (BalanCD) medium (Irvine Scientific) supplemented with 4mM glutamine (Thermo Fisher) were cultured in shake flasks (Corning) at 5% CO_2_, 37°C, and 125 rpm in a shaking incubator (25 mm orbital diameter). For routine maintenance, cells were cultured in a 30 mL working volume and passaged to 0.3 × 10^6^ cells/mL every 4 days. Cells used for rAAV production were cultured in 30 mL working volume in a 125 mL shake flask between passages five and 20. Cell counts and viability were taken using a Cedex HiRes (Roche, Switzerland).

### 2.4 ∣ Transient transfection and rAAV vector production

Complexes of PEIpro (Polyplus) and the three rAAV production plasmids, pAdDeltaF6 (pHelper), pAAV2/2 (pRepCap), and pAAV-CMV-GFP (pGoI) were used to initiate vector production in cultures resuspended with fresh BalanCD media. The cell density at transfection (2 × 10^6^ cells/mL) and plasmid molar ratio (pHelper: pRepCap: pGoI = 2:1.5:1) were fixed based on previous studies (Grieger et al., 2016; Gu et al., 2018). Complexes were prepared by diluting plasmids in fresh BalanCD media, adding PEIpro, incubating the complexes, and adding the complexes to each production flask. The PP setpoint ranges were: PEI:DNA ratio, 1–5; total DNA mass, 0.5–1.5 pg/cell; cocktail volume, 5–17% of the final cell culture volume (CV) following complex addition; and incubation time, 5–30 min. Complexation and transfection were performed in three rounds containing 11 flasks each. Three flasks per round were replicated center points. Production flasks were cultured for 3 days before batches were terminated. Daily samples were taken to monitor cell count and viability.

### 2.5 ∣ rAAV harvest

Following the termination of rAAV production, 1 mL cell culture aliquots (cells and supernatant inclusive) were placed in 1.5 mL centrifuge tubes to harvest the rAAV for immediate PQA analysis or stored at −80°C for future analysis. rAAVs were harvested from each sample by three rounds of freeze thaw. During each round, freezing was done in an ethanol and dry ice slurry for 2 min and thaw was performed in a 37°C water bath for 3 min followed by vortexing to further break open the HEK293 cells. Samples were then treated with 5.5 mM MgCl_2_ (Sigma-Aldrich) and 30 U/mL Benzonase (Sigma Aldrich) for 1 h. Cell debris was spun out at 4100 g and 4°C for 40 min, and samples were transferred to a new 1.5 mL tube for immediate analysis or −80°C storage.

### 2.6 ∣ qPCR for single-target vector genome titer measurement

The harvested rAAV samples were treated with DNase I in DNase Reaction Buffer (New England Biolabs) and incubated for 1 h at 37°C following the manufacturer's instructions. Samples were then treated with 20 U/mL proteinase K (New England Biolabs) and incubated for 1 h at 55°C, followed by a 10 min inactivation step at 95°C. Each sample was then diluted x125 in water by serial dilution (x5, x25, x125). Six dilutions of rAAV5 standard material (MassBiologics) were prepared identically to the rAAV2 samples for use as a standard curve for unknown sample quantification (Yu Wang et al., 2020; Werling et al., 2015). Negative controls (no primer/probe mixture), no template controls (NTCs), and positive controls (pAAV-CMV-GFP linearized with HindIII) were also included on each plate. qPCR analysis targeted the transgene (eGFP) using a PrimeTime qPCR probe assay (Integrated DNA Technologies) detailed in Supporting Information: Table S2. Each 20 μL qPCR reaction contained 10.0 μL TaqMan^™^ Universal PCR Master Mix (ThermoFisher Scientific), 1.0 μL primer/probe mix (500 nM primers/250 nM probe), 6.0 μL molecular biology grade water, and 3.0 μL x125 diluted sample. Reactions were run on a CFX96 Real-Time PCR Detection system (Bio-Rad) for 20 s at 95°C followed by 39 cycles of two-step thermal cycling for 3 s at 95°C and 30 s at 60°C with a plate read. Data analysis was performed in Microsoft Excel. Regression analysis for plots of the log_10_ rAAV5-eGFP standard concentration versus the associated quantification cycle (C_q_) was performed to generate the standard curve. These equations were then used to determine the VG titer.

### 2.7 ∣ ELISA for CP titer quantification

rAAV2 CP titers were measured on the harvested lysate samples using a Progen AAV2 titration ELISA kit 2.0 R (Progen Biotechnik GMBH) per the manufacturer's instructions. Crude lysate rAAV2 samples were serially diluted from 5x-125x such that the final concentration was within the Progen AAV2 empty capsid standard curve range (3.91E7–2.50E9 capsids/mL). All serially diluted kit standards, unknowns, and blanks were run in duplicate. Following assay execution, the plate was read with a Synergy^™^ LX Multi-Mode microplate reader (BioTek) at 450 and 650 nm. The sample optical density (OD) values at 650 nm were subtracted from the 450 nm measurements to perform background subtraction for all samples and standards. The rAAV2 CP titer of unknown samples was calculated from a four-parameter logistic (4-PL) standard curve generated from the subtracted OD measurements of the serially diluted kit controls in Microsoft Excel. Calculated CP titer measurements for unknowns were then corrected by a factor of x2 to account for matrix effects from the lysate. The correction factor was determined by running HEK293 null cell lysate spiked with rAAV2 reference material (Vigene Biosciences) at multiple dilutions and comparing the values calculated from the ELISA 4-PL standard curve with the published concentrations from Vigene (Supporting Information: Figure S1, Supporting Information: Table S3).

### 2.8 ∣ Multiplex VG titer analysis by qPCR

rAAV2 crude lysate samples used for multiplex genome titer analysis were treated with DNase I and Proteinase K identically to the samples analyzed for single-plex vector genome titer. Samples were diluted 5x in molecular biology grade water after treatment (50x total dilution) before multiplex qPCR. pGoI was linearized with DraIII (New England Biolabs) per the manufacturer's instructions, desalted with the QIAqiuck PCR Purification Kit (Qiagen), and the concentration was quantified by UV absorbance (A260) on a DS-11 FX+ (DeNovix) before serial dilution to generate a 7-point standard curve (10^9^–10^3^ copies of pGoI per reaction). Multiplex qPCR analysis targeted the cytomegalovirus (CMV) promoter, eGFP, and the human β-globin polyadenylation signal (polyA tail) with PrimeTime qPCR probe assays containing Cy5, FAM, and HEX probes, respectively (Supporting Information: Table S2). Each 20 μL qPCR reaction contained 10.0 μL TaqMan Fast Advanced qPCR Master Mix (ThermoFisher Scientific), 1.0 μL each of all three primer/probe mixes (900 nM primers/250 nM probe), 4.0 μL molecular biology grade water, and 3.0 μL sample or standard. Reactions were run in quadruplicate in white, 384-well, skirted qPCR plates (ThermoFisher Scientific) on a CFX384 Touch Real-Time PCR Detection System (BioRad). The thermal cycling protocol treated samples at 50°C for 2 min (1 cycle), denatured samples at 95°C for 20 s (1 cycle), and performed two-step cycles at 95°C for 3 s and 60°C for 30 s followed by a plate read (40 cycles). All multiplex qPCR data analysis was performed in Python 3. Regression analysis for plots of the log_10_ plasmid standard concentration versus the associated C_q_ for each fluorophore (Cy5, FAM, HEX) was performed to generate standard curves. These equations were then used to determine the concentration of each target (CMV promoter, eGFP, and polyA tail) in all rAAV2 unknown samples.

### 2.9 ∣ Processing of qPCR, ELISA, and multiplex qPCR data for input into MODDE^®^ as PQA values

VG titer values calculated using the rAAV5-eGFP standard curves were used directly as the VG titer output ($Y_{1}$) in MODDE^®^ (Supporting Information: Figure S2). The VG titers for eGFP obtained with qPCR (Supporting Information: Figure S2) and the corrected CP titer values measured by ELISA (Supporting Information: Figure S3) and were used to calculate the VG:CP titer ratio output ($Y_{2}$). Multiplex VG titer results were normalized by eGFP concentration to enable 2D titer comparisons by location on the genome. The normalized polyA concentration values were used as the 2D VG titer ratio output ($Y_{3}$) for the model because they showed the widest variability between DoE conditions (Supporting Information: Figure S4). Minimum, target, and maximum values of each PQA were then specified based on physical limitations of the system and understanding of its behavior using the collected qPCR and ELISA data sets.

### 2.10 ∣ Establishment of models for each PQA

Model fitting was performed in MODDE^®^. PQAs were modeled with the following quadratic polynomial (Equation 1) so that interaction terms could be included to optimize model fit and predictive capability:

(1) $$Y=a_{0}+a_{1}x_{1}+a_{2}x_{2}+a_{3}x_{3}+a_{4}x_{4}+a_{12}x_{1}x_{2}+a_{13}x_{1}x_{3}+a_{14}\;\;x_{1}x_{4}+a_{23}x_{2}x_{3}+a_{24}x_{2}x_{4}+a_{34}x_{3}x_{4}+a_{11}x_{1}^{2}+a_{22}x_{2}^{2}+a_{33}\;\;x_{3}^{2}+a_{44}x_{4}^{2}$$

In Equation 1, Y is a measured PQA; $a_{0}$ is an intercept; $a_{1}$ to $a_{44}$ are regression coefficients calculated from values of Y observed for all 33 DoE conditions; and $x_{1}$, $x_{2}$, $x_{3}$, and $x_{4}$, are the coded levels of the input PPs. The terms $x_{1}x_{2}$, $x_{1}x_{3}$, $x_{1}x_{4}$, $x_{2}x_{3}$, $x_{2}x_{4}$, $x_{3}x_{4}$, and $x_{i}^{2}\;(i=1,\;2,\;3,\;4)$ represent the interaction and quadratic terms, respectively, for the PPs. PLS analysis was used to estimate the coefficient terms in the models to maximize the correlation between PPs and PQAs in the projected space. Nonsignificant model terms (*p* > 0.05) were then removed using the MODDE^®^ auto tuner until no further improvement in model fit was observed.

### 2.11 ∣ DS determination, robustness testing, and model verification

The optimizer tool in MODDE^®^ was used to perform a limit optimization to reach a solution where the three PQAs were within the minimum and maximum specifications set during experimental design (Table 1). Monte Carlo simulations were run to establish the DS for PP operating ranges and find optimal setpoints in which the PQA specifications were met. The acceptance limit was set to 1% POF, equivalent to 10,000 defects per million opportunities; resolution was set to 16 discrete blocks; and 50,000 simulations were performed in each block per response. Model error was included in the DS plot. MODDE^®^ was subsequently used to search for the robust setpoint that would maximize the distance from the acceptance boundaries in the DS while still maintaining a low POF. Six verification experiments were then carried out in biological duplicate to verify the model predictive power and the DS boundaries. Of these six transfection conditions, two were within and four were outside the DS.

## 3 ∣ RESULTS

### 3.1 ∣ Fitting of mathematical models for PQAs

Equation 1 was fit for each PQA by PLS regression using the data entered in MODDE^®^ (Supporting Information: Table S1). Transformation of each data set was performed as needed to ensure all response data distributions were normal to improve predictive capability. Logarithmic transformation was applied to the VG titer and 2D VG titer ratio data, while a transformation for the VG:CP ratio data was not needed. Coefficients for model terms were estimated using PLS regression, and nonsignificant terms (*p* > 0.05) were removed until optimal model fit was observed (Supporting Information: Table S4) for VG titer ($Y_{1}$), VG:CP ratio ($Y_{2}$), and 2D VG titer ratio ($Y_{3}$) (Equations 2-4).


(2) $$Y_{1}=12.69-0.29x_{1}-0.26x_{2}-0.03x_{3}+0.03x_{4}-0.15x_{1}x_{2}\;\;+0.10x_{3}x_{4}$$

(3) $$Y_{2}=0.19-0.03x_{1}-0.12x_{2}+0.04x_{3}-0.05x_{4}-0.06x_{1}x_{4}\;\;-0.04x_{2}x_{3}+0.06x_{2}^{2}$$

(4) $$Y_{3}=0.37+0.03x_{1}+0.01x_{2}-0.03x_{4}-\;\;0.05x_{1}x_{2}-0.01x_{2}^{2}\;\;+0.03x_{4}^{2}$$

All models contain a constant term, at least three first order terms, and at least two second order terms. The models for VG titer and VG:CP ratio contain significant terms that include all four PP inputs. However, the model for 2D VG titer ratio does not contain a significant term that includes cocktail volume ($X_{3}$).

### 3.2 ∣ Contour plot and interaction analysis for PQAs

**FIGURE 1:**
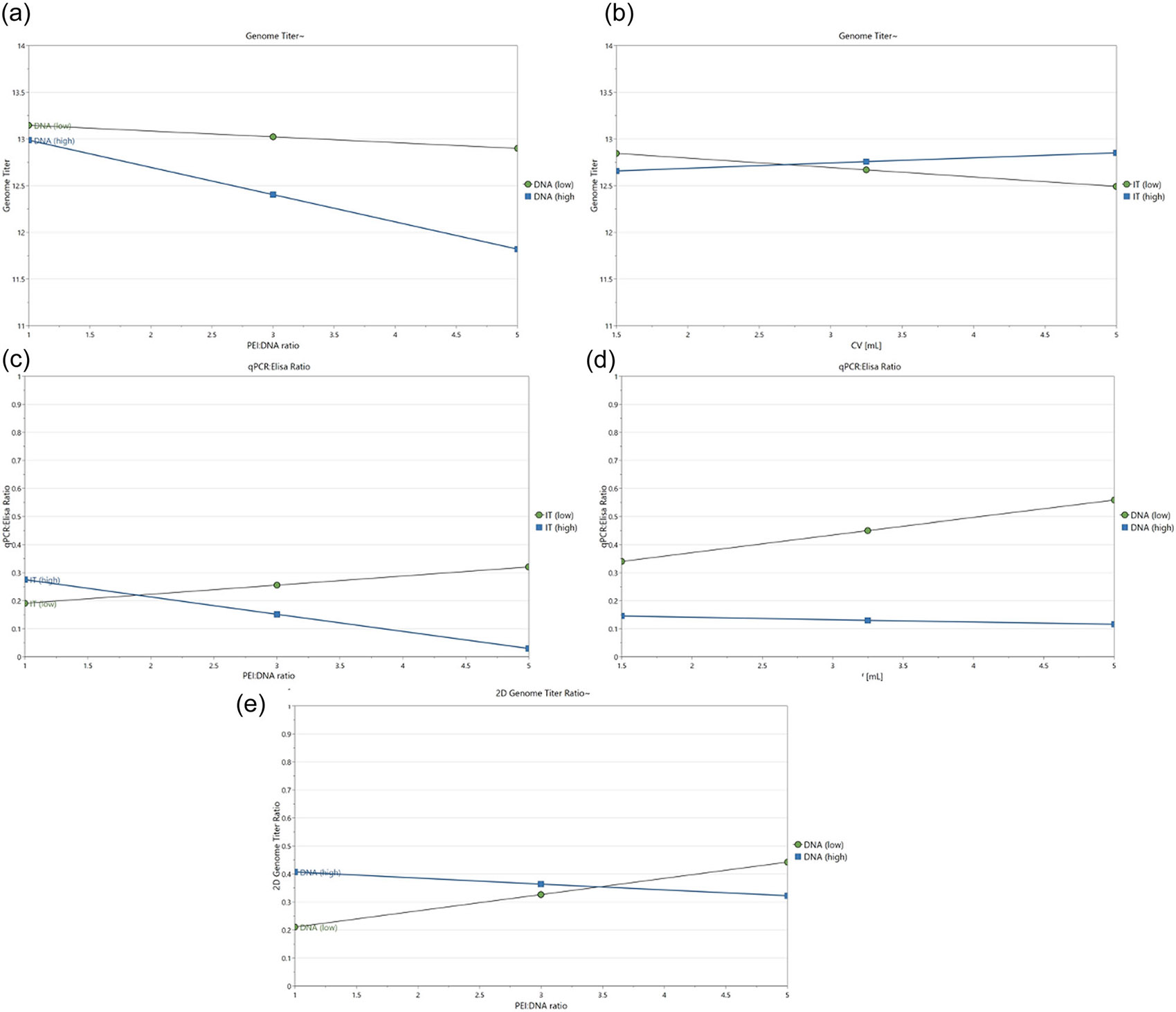
Interaction plots depicting second order interactions between input PPs and output PQAs. Transformed VG titer was affected by interactions between (a) PEI:DNA ratio and total DNA/cell (DNA) and (b) cocktail volume (CV) and incubation time (IT). VG:CP) ratio was affected by interactions between (c) PEI:DNA ratio and incubation time (IT) and (d) cocktail volume and total DNA/cell (DNA). 2D VG titer ratio was affected by interactions between (e) PEI:DNA ratio and total DNA/cell (DNA). CP, capsid particle; PEI, polyethyleneimine; PQA, product quality attributes; VG, viral genome.

**FIGURE 2:**
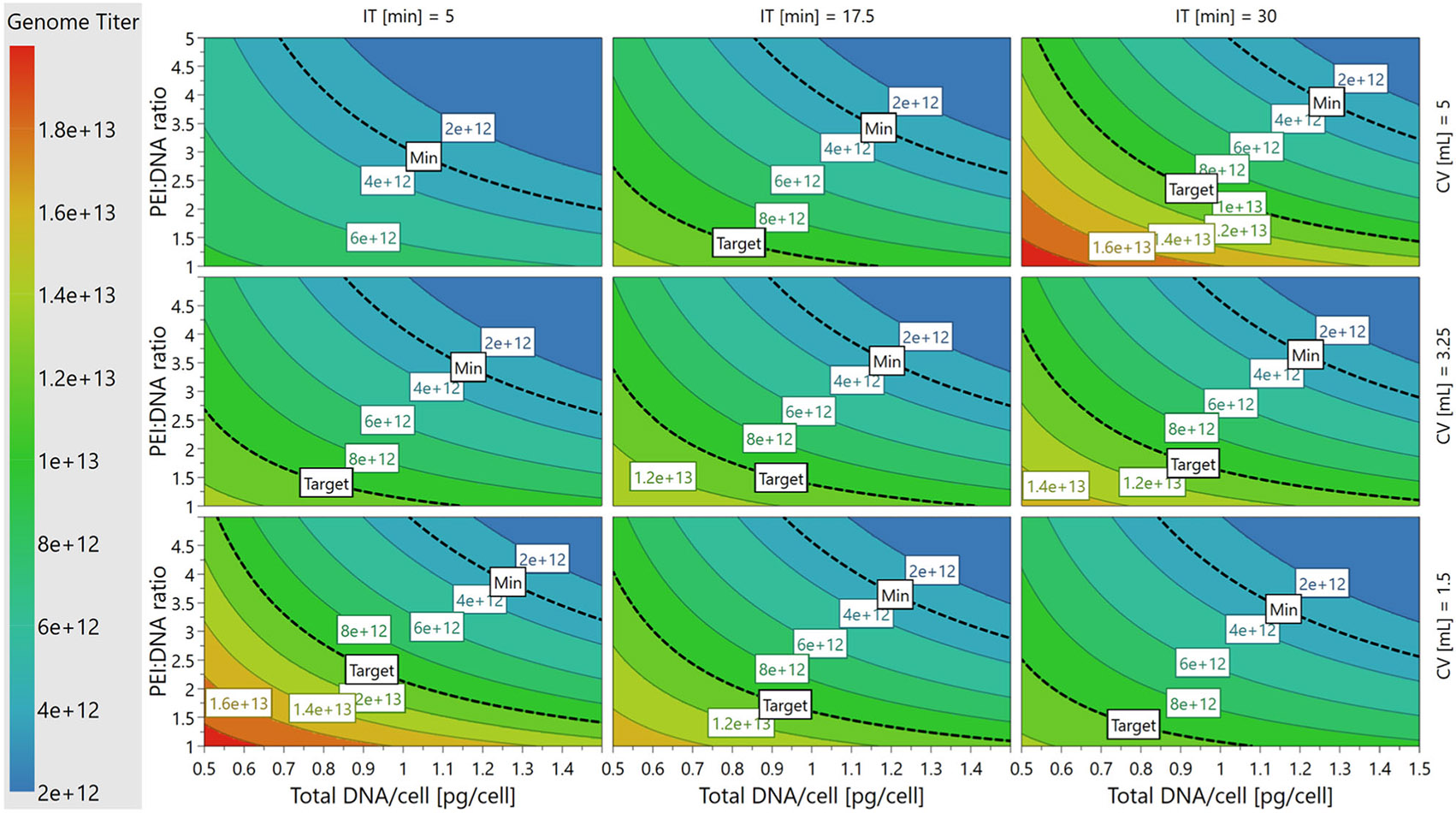
4D contour plot showing the relationship between Genome Titer (VG titer) and PPs. 4D contour plot showing how non-transformed VG titer changes with the values of all four PPs. The minimum VG titer specification, 3E + 12 vg/L, is marked on all sub-plots. 2D, two-dimensional; 4D, Four-dimensional; PP, process parameters; VG, viral genome.

**FIGURE 3:**
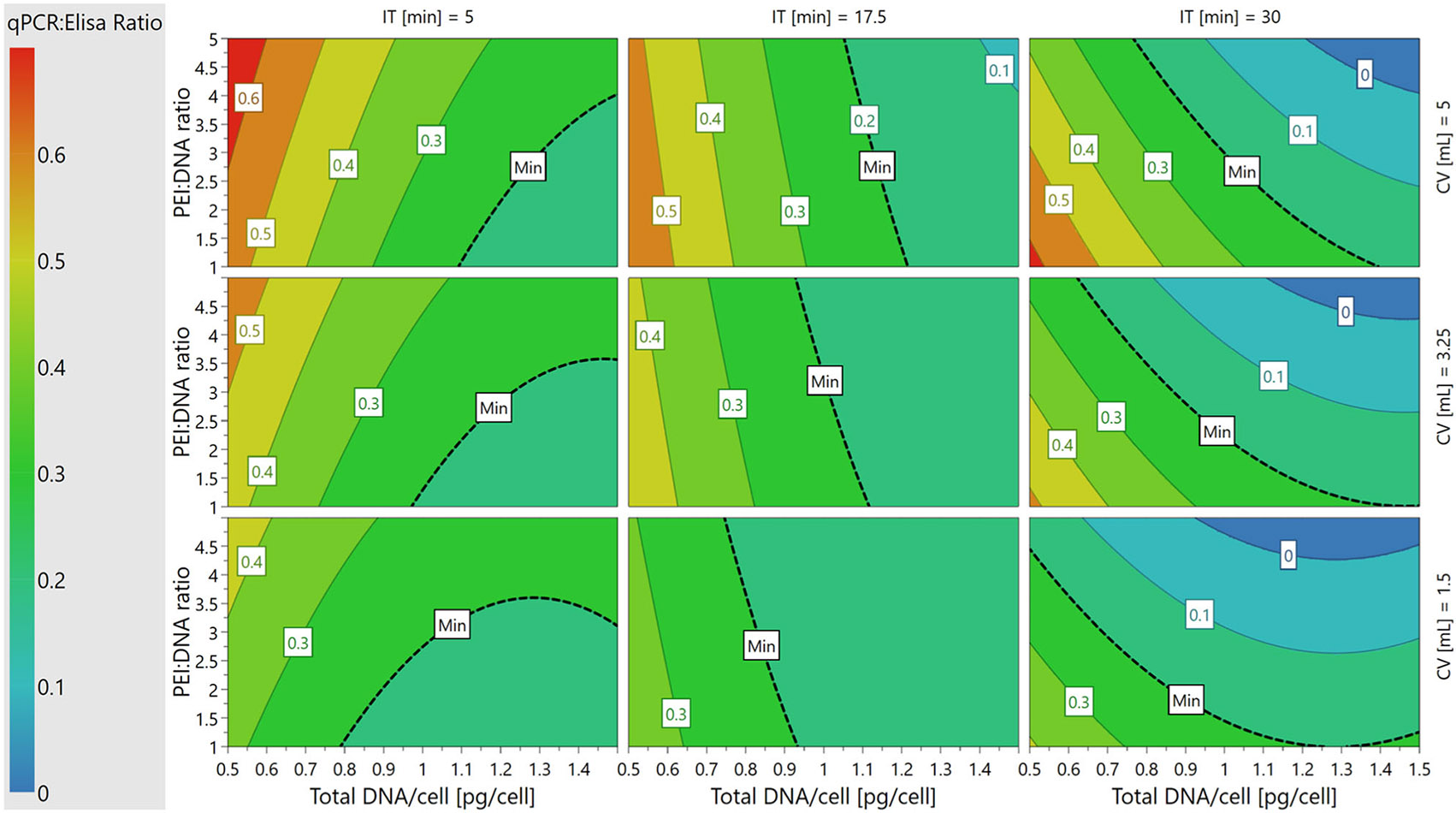
4D contour plot showing the relationship between qPCR:ELISA (VG:CP) ratio and PPs. 4D contour plot showing how VG:CP titer ratio changes with the values of all four PPs. The minimum VG:CP ratio specification, 0.2, is marked on all sub-plots. 4D, Four-dimensional; CP, capsid particle; ELISA, enzyme-linked immunosorbent assay; PP, process parameters; qPCR, quantitative polymerase chain reaction; VG, viral genome.

**FIGURE 4:**
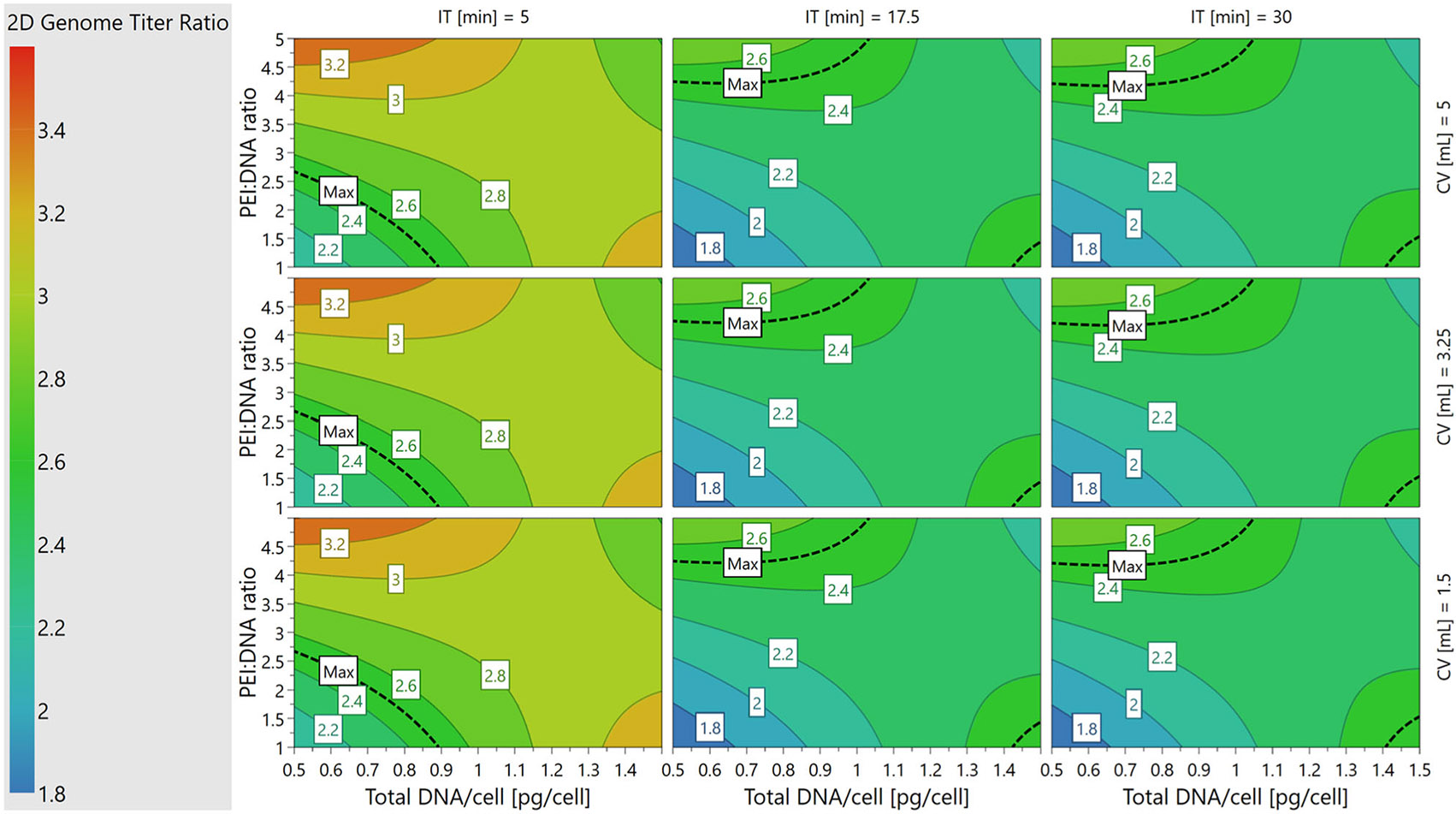
4D contour plot the relationship between 2D Vector Genome Titer Ratio (2D VG titer ratio) and PPs. 4D contour plot showing how non-transformed 2D VG titer ratio changes with the values of all four PPs. The maximum 2D VG titer ratio specification, 2.5, is marked on all sub-plots. 2D, two-dimensional; 4D, four-dimensional; PP, process parameters; VG, viral genome.

Response surface analysis was performed in MODDE^®^ using interaction (Figure 1) and contour plots (Figures 2-4). Four-dimensional (4D) contour plots were generated using Equations 2-4 to visualize the trends that each PQA followed in response to changes in the PPs. Because our system had four PP inputs, the 4D contour plots enabled visualization of how a PQA on its native, non-transformed scale responded to all PPs in one visual containing nine 2D sub-plots in a 3 × 3 grid. Each individual contour plot shows the response for PEI:DNA ratio versus total DNA/cell as continuous variables, and these plots are tiled for low, medium, and high values of incubation time and cocktail volume. Interaction plots show how second order PP interactions affect PQA values on the transformed scale used for model fitting. One PP is plotted continuously on the x-axis, trends for high/low levels for a second PP are plotted as lines, and the other two PPs are fixed at their averages.

#### 3.2.1 ∣ Influence of PP inputs on VG titer

We observed that VG titer was highest when the PEI:DNA ratio and total DNA/cell were kept at lower setpoints, regardless of cocktail volume or incubation time (Figure 2). Minimizing the total mass of PEI added to the complexation reaction also had a positive correlation with resulting VG titers (Supporting Information: Figure S5A). The second order interactions between PEI:DNA ratio and total DNA/cell (Figure 1a) provide further insight into these trends. VG titer was highest when PEI:DNA ratio was lowest for both the low and high DNA amount setpoints. Interestingly, the rate at which VG titer dropped as PEI:DNA ratio increased is higher for the high DNA setpoint versus the low DNA setpoint, which indicates there was a cumulative negative effect of adding larger amounts of PEI and DNA to the complexation reaction and subsequent cell culture.

The highest VG titers (>1.4E13 vg/L) on the individual 2D contour plots, depicted by the red and orange regions in Figure 2, occurred when cocktail volume and incubation time were both at the lowest or highest setpoints. This corresponds to complexing PEI and plasmid DNA in 1.5 mL for 5 min or in 5 mL for 30 min. These results suggest that the rate at which complex size increased depended on cocktail volume, specifically that PEI and plasmids reached a complex size that maximizes VG titer more quickly in smaller volumes versus larger volumes. We observed that VG titer decreases with cocktail volume for the low incubation time setpoint, while titer increases with cocktail volume for the high incubation time setpoint (Figure 1b).

#### 3.2.2 ∣ Influence of PP inputs on VG:CP ratio

VG:CP ratio always had a negative correlation with the total DNA/cell, regardless of incubation time (Figure 3). However, the relationship between VG:CP ratio and PEI:DNA ratio was incubation time dependent. At 5 min incubation time, the VG:CP ratio had a positive correlation with PEI:DNA ratio, but at 17.5 and 30 min incubation times, there was a negative correlation between VG:CP ratio and PEI:DNA ratio (Figure 3). Visualizing the second order interactions between PEI:DNA ratio and incubation time further detailed these trends. At the low incubation time setpoint, increasing the amount of PEI had a modest positive effect on the VG:CP ratio (Figure 1c). This observation suggests that using larger amounts of PEI at shorter incubation times allowed sufficient amounts of each plasmid to be incorporated into the complexes to enable the formation of full capsids. Conversely, at the high incubation time setpoint, increasing the amount of PEI decreased the VG:CP ratio more drastically. At these longer incubation times, the plasmid DNA likely had enough time to form complexes conducive to effective transfection with less total PEI, making the increase in PEI mass more detrimental to cell health than it was useful in promoting full capsid formation (Supporting Information: Figure S5B/C, Supporting Information: Table S1).

Cocktail volume also had an impact on the VG:CP ratio, as the highest ratios (>0.5) on the individual 2D contour plots, depicted by the red and orange regions in Figure 3, occur over the widest range of input conditions at the highest cocktail volume setpoint (5 mL). It is also worth noting that the cocktail volume and total DNA/cell had a second order relationship (Figure 1d). At the low total DNA/cell setpoint, the VG:CP ratio increased with cocktail volume, but at the high DNA/cell setpoint, VG:CP ratio decreased as cocktail volume increased. These trends are the opposite of what was observed for VG titer, indicating that there are likely tradeoffs to be made when setting optimal setpoints for cocktail volume and total DNA/cell to maximize both VG titer and VG:CP ratio.

#### 3.2.3 ∣ Influence of PP inputs on 2D VG titer ratio

The multiplex qPCR assay for VG titer enabled population-level analysis of the genomes in each sample as an alternative to published two-plex ddPCR assays for single-genome level analysis (Furuta-Hanawa et al., 2019; Zanker et al., 2022). Use of the polyA:EGFP titer ratio as the 2D VG titer ratio allowed us to model how the four transfection PPs may affect partial capsid formation. A 2D VG titer ratio as close to 1 as possible is ideal, as was seen for plasmid DNA controls, and could indicate that a larger percentage of packaged genomes are full-length (Supporting Information: Figure S4).

The 2D VG titer ratio was minimized when PEI:DNA ratio and total DNA/cell were kept at lower setpoints across all combinations of cocktail volumes and incubation times (Figure 4). Interestingly, these trends matched those that were ideal for maximizing VG titer (Figure 2), and for which VCD and cell viability were highest (Supporting Information: Table S1). The necessity of minimizing PEI:DNA ratio and total DNA/cell to minimize 2D VG titer ratio was more pronounced at the shortest incubation time (5 min) versus longer incubation times (17.5 and 30 min). The lowest values of 2D VG titer ratio (<2) on the individual 2D contour plots, depicted by the blue regions in Figure 4, occurred when incubation times were kept longer at 17.5 or 30 min.

The interaction plot depicting how PEI:DNA ratio and total DNA/cell affect 2D VG titer ratio showed further nuances in the relationships between these variables (Figure 1e). At the low DNA/cell setpoint, the 2D VG titer ratio increased with PEI:DNA ratio, while at the high DNA/cell setpoint, 2D VG titer ratio decreased as PEI:DNA ratio increases. The lowest 2D VG titer ratios are reached when PEI and DNA amounts are both low, but decrease again over the contours when both of these inputs are high.

### 3.3 ∣ DS establishment, setpoint optimization, and model verification

**FIGURE 5:**
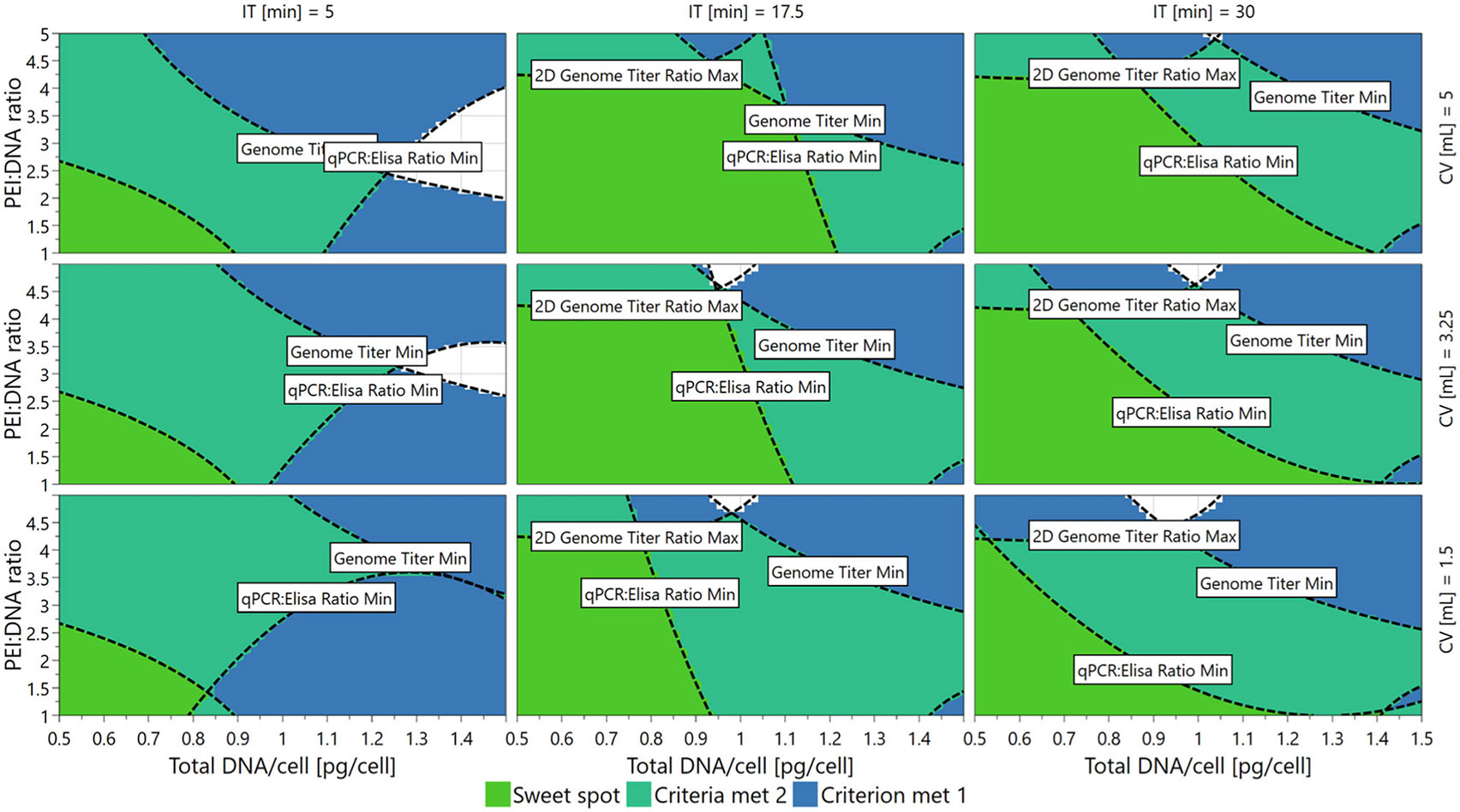
Sweet spot plots showing PP ranges in which the PQAs are within specifications. The 4D sweet spot plot mimics the layout of the 4D contour plots and shows regions where all three (green), two (teal), one (blue), or zero (white) PQA specifications are met based on the established models. Genome Titer = VG Titer, qPCR:ELISA ratio = VG:CP ratio, 2D Genome Titer Ratio = 2D VG Titer Ratio. 2D, two-dimensional; 4D, four-dimensional; CP, capsid particle; ELISA, enzyme-linked immunosorbent assay; PPs, process parameters; PQAs, product quality attributes; qPCR, quantitative polymerase chain reaction; VG, viral genome.

We next sought to establish and validate the DS for PP ranges in which the PQA specifications set during experimental design were met (Table 1). To visualize the PP ranges in which all three, two, one, or zero PQA specifications could be met based on the established models, a 4D sweet spot plot was generated that contained nine 2D sub-plots in a 3 × 3 grid (Figure 5). Each individual sweet spot plot showed the response regions for PEI:DNA ratio versus total DNA/cell on continuous axes, and these plots are tiled for low, medium, and high values of incubation time and cocktail volume. Regardless of incubation time or cocktail volume, all three PQA specifications were met when PEI:DNA ratio and total DNA/cell were kept at low setpoints as shown by the green regions in Figure 5. Conversely, transfecting with a high PEI:DNA ratio and high total DNA/cell led to one or none of the PQA criteria being met as shown by the blue and white regions in Figure 5, respectively.

**FIGURE 6:**
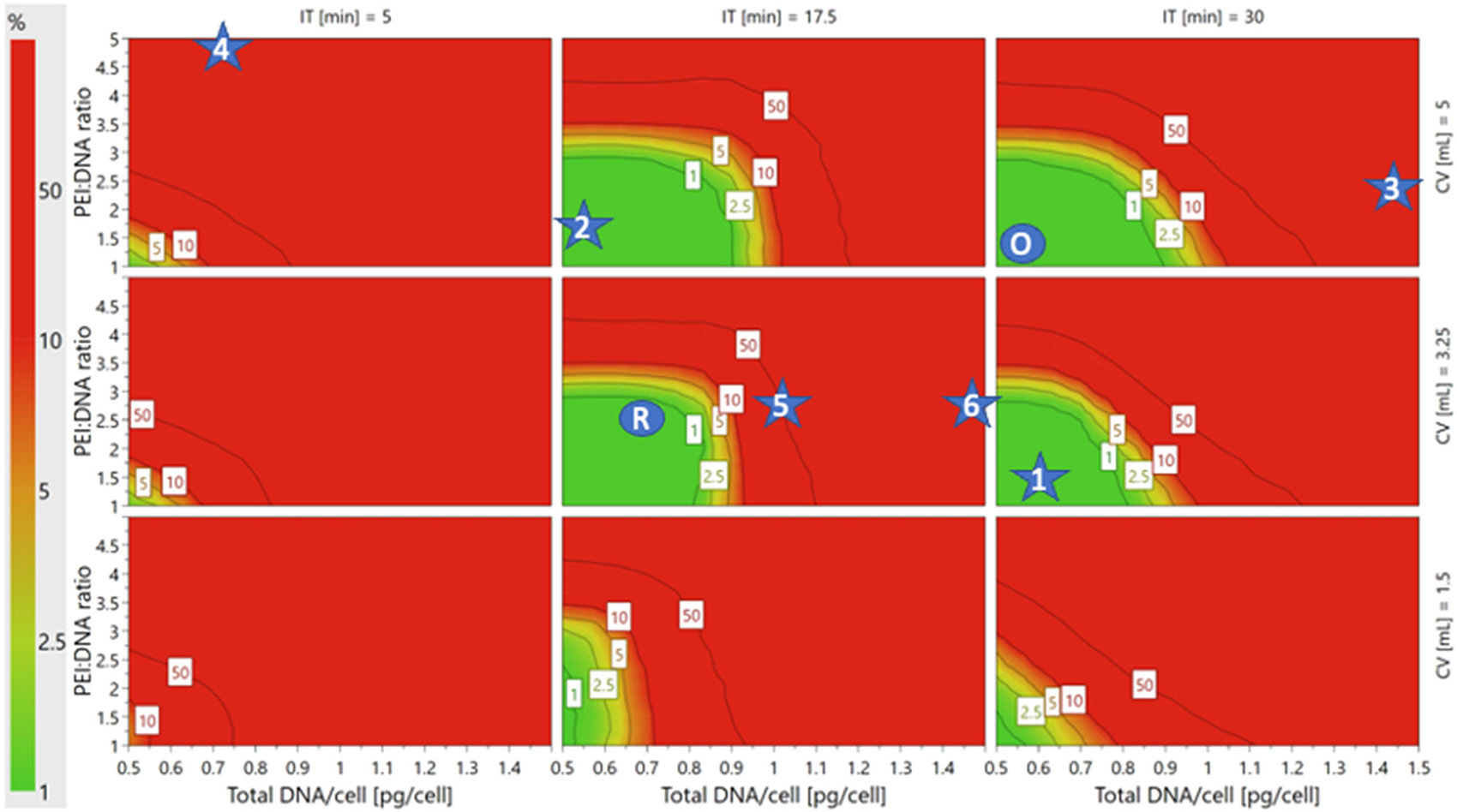
Design space (DS) plots showing the probabilities of failure that the PQAs specifications will not be met in associated PP ranges. The 4D DS plots have the same layout as the 4D sweet spot plots and depict the PP space where all PQAs will be met with <1% POF in green. The optimal (O) and robust (R) setpoints are marked with blue circles, and the six verification conditions are marked with blue stars. 4D, Four-dimensional; POF, probability of failure; PPs, process parameters; PQAs, product quality attributes.

While the sweet spot plots highlighted the PP ranges in which where all the PQAs are within the ranges specified during experimental design, they did not show the probabilities that PQA specifications would be met across the PP space. To evaluate where in the 4D PP space the models are most likely to meet all three PQAs, a 4D DS plot was generated based on data from the Monte Carlo simulations to predict model failure (Figure 6). The regions that met a≤1% POF acceptance criteria were defined as the DS, as shown in green in Figure 6. The DS spans regions where PEI:DNA ratio and total DNA/cell are both low, as in the sweet spot plots. However, DS regions on each 2D plot were smaller than green regions on the sweet spot plots. This indicated that not all PP setpoints in the green regions of the sweet spots were acceptably robust. Interestingly, the size of the DS region on each 2D plot changes with incubation time and cocktail volume. Larger DS regions occur at combinations of middle or high setpoints of incubation time (17.5 or 30 min) and cocktail volume (3.25 or 5 mL). At low incubation times (5 min) and cocktail volumes (1.5 mL), the DS is small or nonexistent, which suggests that the balance between PPs that is required to achieve optimal control over PQAs is more easily disrupted or cannot be met for processes using these setpoints.

**FIGURE 7:**
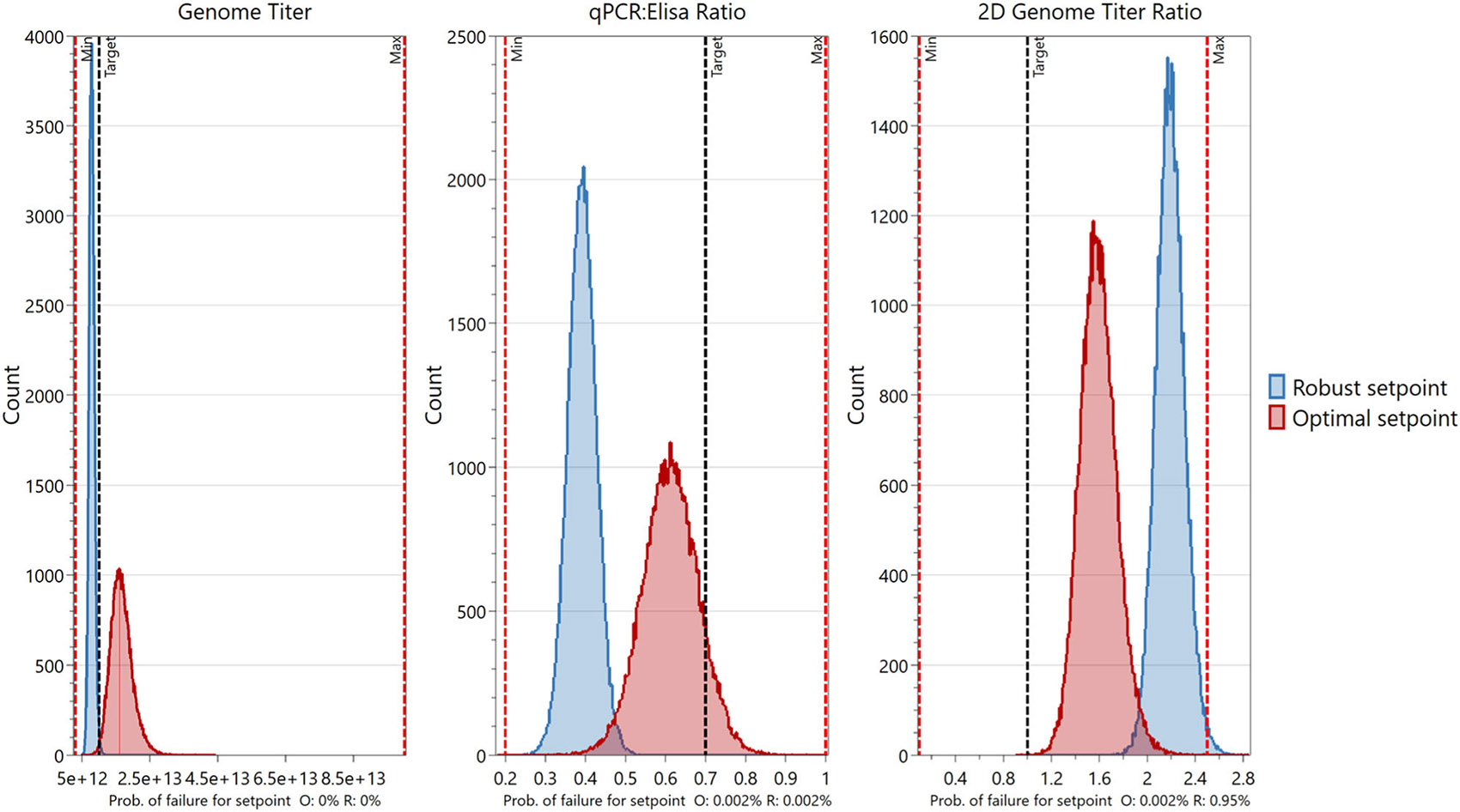
Plots depicting the predicted PQA values for the optimal and robust PP setpoints. Monte Carlo simulations were performed in MODDE^®^ to identify the optimal (O, in red) and robust (R, in blue) PP setpoints. Sub-plots show the number of simulations versus the PQA value. Probabilities of failure for each setpoint per PQA are shown below the x-axis of each sub-plot. PPs, process parameters; PQAs, product quality attributes.

The Monte Carlo simulations also identified the optimal and robust setpoints (Figure 7) that had the most desirable PQA output values and lowest POF, respectively. PP values, PQA measurements, and POFs associated with these setpoints are shown in Table 2, and their locations on the 4D DS plot are marked by blue circles in Figure 6. The robust setpoint occurs at the middle incubation time and cocktail volume (17.5 min and 3.25 mL) while the optimal setpoint occurs at the high incubation time and cocktail volume (30 min and 5 mL). While both the robust and optimal setpoints met the 1% POF requirement, the PQA distributions for the robust setpoint simulations were noticeably tighter than those for the optimal setpoint. Notably, the robust setpoint distribution was narrowest and closest to specification for VG titer, and the VG titer model had the highest model fit (*R*^2^) and predictability (*Q*^2^) versus the models for VG:CP ratio and 2D VG titer ratio (Supporting Information: Figure S6).

To confirm the predictive capability of the model and validate the DS, six verification conditions were tested, as indicated by numbered blue stars in Figure 6. The cultures for the two conditions within the DS that had a 1% predicted POF produced rAAV with VG titers, VG:CP ratios, and 2D VG titer ratios within specification. As expected, the four cultures run with conditions outside of the DS did not meet all three PQA specifications (Table 3). All four conditions outside of the DS met the 2D VG titer ratio specification, only condition 6 met the VG titer specification, and none of the conditions met the VG:CP ratio specification. Our verification experiments confirmed that we were able to define PPs to control rAAV2 VG titer, VG:CP ratio, and 2D VG titer ratio resulting from transient transfection of HEK293 cells using the described DoE methodology.

## 4 ∣ DISCUSSION

This work has established transient DNA complexation PP operating ranges in which the three studied PQAs, VG titer, VG:CP ratio, and 2D VG titer ratio, are met with the lowest POF. Performing complexation at the middle-high ranges for incubation time (17.5–30 min) and cocktail volume (3.25–5 mL), and low-middle ranges for PEI:DNA ratio (1:1–3:1) and total DNA/cell (0.5–1 pg/cell), enables specifications for all three PQAs to be met at <1% POF with the widest PP range tolerances (Figure 6). When our results are compared to the existing literature focused solely on maximizing VG titer, we see that our optimal settings for PEI:DNA ratio and total DNA/cell match those of several studies, while our best settings for incubation time and cocktail volume are generally higher.

Specifically, multiple studies report that a reagent:DNA ratio of 2:1 or 3:1 ratio is optimal for maximizing VG titer (Grieger et al., 2016; Gu et al., 2018; Zhao et al., 2020), which is in the DS range that optimizes our three PQAs. We also noted that our optimal total DNA/cell (0.5–1 pg/cell) matches that of two other studies (Grieger et al., 2016; Gu et al., 2018), but there are other studies that transfect with two to threefold more DNA (1.5–3 pg/cell) (Guan et al., 2022; Zhao et al., 2020). Interestingly, comparison of the VCD at transfection between studies shows that cultures with lower starting VCDs generally require more DNA/cell to reach optimal VG titers. Previous literature has focused less on optimizing cocktail volume or incubation time to maximize VG titer, and most studies have complexed in 5–10% CV for 10–15 min (Grieger et al., 2016; Gu et al., 2018; Zhao et al., 2020). In our study, complexing in 17% CV for 30 min or 11% CV for 17.5 min was best for optimizing all three PQAs. Operating the complexation process at low PEI:DNA ratio and low total DNA/cell setpoints also helped maintain optimal cell culture health in our study. Regression analysis showed that the total mass of PEI introduced during transfection (PEI:DNA ratio x total DNA/cell; $X_{1}\;x\;X_{2}$) has a strong negative correlation (*R*^2^ > 0.9) with the log of VG titer, VCD, and cell viability, (Supporting Information: Figure S5). Specifically, low cell viabilities were observed to occur because of PEI-mediated toxicity, as all conditions with a PEI amount > 6 pg/cell had culture viabilities < 42%. These runs had VG titers ≤ 1E12 VG/L. The upper PEI mass limit cells can tolerate without large drops in viability may be ~3–5 pg/cell since conditions with PEI amount of 3 pg/cell yielded moderate to high cell viability (74.7–98.4%). While the cytotoxicity of hydrolyzed or deacetylated forms of linear PEI such as PEIpro and PEI MAX has also been observed in other studies (Delafosse et al., 2016; Grieger et al., 2016; Gu et al., 2018; Zhao et al., 2020), these reagents are used for rAAV production because they are able to bind and transfer DNA more efficiently than regular linear PEI (Kadlecova et al., 2012; Thomas et al., 2005). We also observed that total DNA has a moderate negative correlation with VCD (Supporting Information: Figure S7). Taken together, these observations indicate that optimizing the amount of DNA and PEI in tandem can balance the cytotoxicity of transfection reagents with the need for high titer rAAV production. Additionally, designing the complexation process to use less total PEI and plasmid DNA is economically advantageous because both raw materials are expensive to use at commercial scale.

The amount of PEI used for complexation also had a threshold effect on transfection efficiency due to its cytotoxicity. This is evident when looking at the relationship between transfection efficiency and complex size. After excluding cultures that had cell viability < 50% due to transfection with large amounts of PEI (Supporting Information: Figure S8A/B), there is a negative correlation between transfection efficiency and particle size (*R*^2^=0.79) (Supporting Information: Figure S8C). To our knowledge, optimal complex sizes for rAAV production have not been extensively studied in literature and may be cell line dependent, but one study shows that optimal production of lentivirus particles occurred after transfection with complexes 400–500 nm in size (Hu et al., 2021). Complexes that most effectively transfected cells in our study were similarly sized, ranging from 400 to 600 nm (Supporting Information: Figure S8). Previous literature has shown that larger complexes may not enter cells as efficiently due to high degrees of DNA condensation hindering DNA release inside the cells, and formation of oversized polyplexes damaging cell membranes or preventing complexes from entering cells (Han et al., 2009; Kadlecova et al., 2012; van Gaal et al., 2011). When looking at our results by condition instead of overall trends, we observed that run #18 is the best performing condition, as it had the highest VG titer, highest VG:CP ratio, and a relatively low 2D VG titer ratio. Interestingly, this run had a complex size of 570.8 nm, which was within the aforementioned ideal complex size range, and had a high transfection efficiency of 79.75%. The long incubation time (30 min) and high cocktail volume (5 mL) for this run may have allowed for slower formation of complexes with an ideal diameter and minimized aggregate formation.

Although complex size and transfection efficiency were correlated, neither of these parameters had strong correlations with VG titer, VG:CP ratio, or 2D VG titer ratio. Achieving high transfection efficiency is important for initial plasmid transfer, but these results suggest that are other factors related to gene expression following plasmid entry into cells that can be optimized in future studies to enhance control of PQAs related to full, partial, and empty capsid populations. Other recent studies illustrate the need for such investigations, as only a small fraction (~7%) of cells appear to produce a measurable amount of assembled AAV capsid even in systems that show high (>60%) transfection efficiency (Dash et al., 2022), and there is a mismatch between the timelines for capsid synthesis and viral DNA replication conditions in currently used transient production processes that leads to high abundance of empty/partial capsids (Nguyen et al., 2021). A previous DoE study explored how to optimize VG titer by varying plasmid ratios in a system with an optimized pRepCap (Zhao et al., 2020), but the relationship between plasmid ratio/gene expression and other PQAs has not been explored. Future work exploring how further improvements to plasmid designs to optimize AAV/Adenovirus helper gene expression and rAAV genome packaging efficiency relate to plasmid ratio in the DS we established for complexation may reveal further insights related to how to maximize VG titer and VG:CP ratio while minimizing 2D VG titer ratio. Based on our model fits, this could greatly assist with control of VG:CP ratio and 2D VG titer ratio, since they had lower correlations (*R*^2^ values of 0.7 and 0.58, respectively) with complexation parameters than VG titer (*R*^2^ > 0.9) (Supporting Information: Figure S6).

## 5 ∣ CONCLUSIONS

This work demonstrates the utility of using a DoE approach to develop models that establish a PP DS for PEI and DNA complexation for transient rAAV production that optimizes three PQAs: VG titer, VG:CP ratio, and 2D VG titer ratio. Performing complexation at the middle-high ranges for incubation time (17.5–30 min) and cocktail volume (3.25–5 mL), and low-middle ranges for PEI:DNA ratio (1:1–3:1) and total DNA/cell (0.5–1 pg/cell), enables specifications for all three PQAs to be met at <1% POF with the widest PP range tolerances. Verification experiments confirmed model predictive capability, and this work establishes a framework for studying additional PPs and their relationship to PQAs.

## Supplementary Material

supplementary materials

## Figures and Tables

**TABLE 1:** PP inputs and PQA responses in the CCO experimental design and their limits/specifications.

Process parameters	Abbreviation	Units	Low level setting	High level setting
$X_{1}$: PEIpro:DNA mass ratio	PEI:DNA ratio	–	1	5
$X_{2}$: Total DNA mass per cell	Total DNA/cell	pg/cell	0.5	1.5
$X_{3}$: Cocktail volume	CV	mL	1.5	5
$X_{4}$: Incubation time	IT	min	5	30
Product quality attributes		Units		Specification
$Y_{1}$: VG titer		vg/L		≥3E + 12
$Y_{2}$: VG:CP ratio		–		≥ 0.2
$Y_{3}$: 2D VG titer ratio		–		< 2.5

Abbreviations: CCO, central composite orthogonal; CP, capsid particle; CV, culture volume; IT, incubation time; PPs, process parameters; PQAs, product quality attributes; VG, viral genome.

**TABLE 2:** Input PP setpoints and median output PQA values for the optimal and robust setpoints.

	PP conditions				Median PQA value			PQA probability of failure		
Setpoint	$X_{1}$	$X_{2}$	$X_{3}$	$X_{4}$	$Y_{1}$	$Y_{2}$	$Y_{3}$	$Y_{1}$	$Y_{2}$	$Y_{3}$
Optimal	1.0	0.5	5.0	25.0	1.59E13	0.63	1.60	0.00%	0.00%	0.00%
Robust	2.6	0.7	3.8	16.7	7.64E12	0.39	2.16	0.00%	0.00%	0.95%

Abbreviations: PPs, process parameters; PQAs, product quality attributes.

**TABLE 3:** Verification experiment results for the developed design space.

Two-direction verification	Run	Conditions				Probability of failure	Experimental results			All PQA specifications met? (Y/N)
		$X_{1}$	$X_{2}$	$X_{3}$	$X_{4}$		$Y_{1}$	$Y_{2}$	$Y_{3}$	
Inside design space	1	1.25	0.6	3.25	30	1%	8.90E12	0.26	1.23	Y
	2	1.5	0.5	5	17.5	1%	7.51E12	0.39	1.29	Y
Outside design space	3	3	1.5	5	30	70%	1.77E12	Below LOD	1.85	N
	4	4.9	0.7	6.6	2	55%	7.04E12	Below LOD	1.64	N
	5	3	1	3.25	17.5	70%	2.25E12	Below LOD	2.01	N
	6	3	1.5	3.25	17.5	99%	4.45E12	Below LOD	2.20	N

Abbreviations: PPs, process parameters; PQAs, product quality attributes.

## Data Availability

The data that support the findings of this study are provided in the supplemental file.
