# Supplementary material for: Design space determination to optimize DNA complexation and full capsid formation in transient rAAV manufacturing
Source: Biotechnol Bioeng. Author manuscript; Available in PMC 2024 Nov 24. (PMC11585969; doi:10.1002/bit.28508)
Supplement: supplementary materials [file NIHMS2034417-supplement-supplementary_materials.docx]

**Supplementary Information**

**Design space determination to optimize DNA complexation and full capsid formation in transient rAAV manufacturing**

Qiang Fu^1*^, Yong Suk Lee^2*^, Erica A. Green^3^, Yongdan Wang^4^, So Young Park^2^, Ashli Polanco^4^, Kelvin H. Lee^3^, Michael Betenbaugh^5^, David McNally^4,6^, Seongkyu Yoon^4†^

1. Department of Biomedical Engineering and Biotechnology, University of Massachusetts Lowell, Lowell, MA 01854
2. Department of Pharmaceutical Sciences, University of Massachusetts Lowell, Lowell, MA 01854
3. Department of Chemical and Biomolecular Engineering, University of Delaware, Newark, DE 19713
4. Department of Chemical Engineering, University of Massachusetts Lowell, Lowell, MA 01854
5. Department of Chemical and Biomolecular Engineering, Johns Hopkins University, Baltimore, MD 21218
6. MassBiologics, University of Massachusetts Chan Medical School, Mattapan, MA 02126

* Lee and Fu equally contributed to this paper.

† Corresponding author. Correspondence should be addressed to Seongkyu Yoon / 1 University Ave, Lowell, MA 01854 / 978-934-4741 / seongkyu_yoon@uml.edu

Short title: Design space for transient rAAV production

**Table S1:** The central composite orthogonal (CCO) design of experiments (DoE) input process parameters(PPs; blue columns), output product quality attributes (PQAs; orange columns), and supplemental measured process characteristics (grey columns).

| Exp No^1^ | PEI: DNA ratio (X_1_) | Total DNA (X_2_, pg/ cell) | Cocktail Volume  (X_3_, mL) | Incubation Time  (X_4_, min) | VG Titer  (Y_1_, VG/L) | VG:CP Titer Ratio (Y_2_) | 2D VG Titer Ratio (Y_3_) | Day 3 Cell  Viability (%)**^2^** | Average Transfection Efficiency (%)**^3^** | PEI & DNA Complex Size (nm)**^4^** |
| --- | --- | --- | --- | --- | --- | --- | --- | --- | --- | --- |
| 1 | 5 | 0.5 | 1.5 | 5 | 1.33E+13 | 0.42 | 2.82 | 83.40 | 85.95 | 602.20 |
| 2 | 1 | 1.5 | 1.5 | 5 | 1.12E+13 | 0.16 | 3.12 | 77.20 | 77.55 | 911.50 |
| 3 | 1 | 0.5 | 5 | 5 | 3.93E+12 | 0.41 | 1.51 | 94.90 | 71.55 | 367.60 |
| 4**^6^** | 5 | 1.5 | 5 | 5 | 5.54E+11 | 0.00 | 2.30 | 29.90 | 46.20 | 542.10 |
| 5 | 1 | 0.5 | 1.5 | 30 | 2.12E+13 | 0.28 | 1.81 | 88.90 | 67.35 | 926.20 |
| 6 | 5 | 1.5 | 1.5 | 30 | 6.04E+11 | 0.02 | 2.08 | 38.40 | 23.70 | 1130.00 |
| 7 | 5 | 0.5 | 5 | 30 | 1.09E+13 | 0.38 | 2.89 | 84.50 | 84.00 | 615.80 |
| 8 | 1 | 1.5 | 5 | 30 | 9.94E+12 | 0.28 | 3.48 | 75.40 | 72.35 | 929.10 |
| 9 | 3 | 1 | 3.25 | 17.5 | 6.59E+12 | 0.36 | 2.31 | 70.80 | 74.65 | 902.40 |
| 10 | 3 | 1 | 3.25 | 17.5 | 5.83E+12 | 0.27 | 2.32 | 68.80 | 73.70 | 796.70 |
| 11 | 3 | 1 | 3.25 | 17.5 | 6.31E+12 | 0.23 | 2.12 | 69.40 | 74.45 | 779.90 |
| 12 | 1 | 0.5 | 1.5 | 5 | 1.63E+13 | 0.39 | 2.06 | 94.30 | 81.80 | 591.00 |
| 13 | 5 | 1.5 | 1.5 | 5 | 1.12E+12 | 0.39 | 2.33 | 42.40 | 48.30 | 721.70 |
| 14 | 5 | 0.5 | 5 | 5 | 5.31E+12 | 0.78 | 3.64 | 86.40 | 81.35 | 372.90 |
| 15 | 1 | 1.5 | 5 | 5 | 9.10E+12 | 0.21 | 3.71 | 76.50 | 76.45 | 557.00 |
| 16 | 5 | 0.5 | 1.5 | 30 | 7.95E+12 | 0.30 | 2.81 | 86.00 | 61.55 | 910.10 |
| 17 | 1 | 1.5 | 1.5 | 30 | 9.69E+12 | 0.14 | 1.66 | 83.70 | 58.50 | 1389.70 |
| 18 | 1 | 0.5 | 5 | 30 | 2.75E+13 | 0.84 | 1.88 | 94.40 | 79.75 | 570.80 |
| 19 | 5 | 1.5 | 5 | 30 | 1.30E+12 | 0.05 | 2.38 | 43.20 | 45.70 | 899.50 |
| 20 | 3 | 1 | 3.25 | 17.5 | 5.34E+12 | 0.16 | 2.67 | 74.20 | 68.75 | 748.90 |
| 21 | 3 | 1 | 3.25 | 17.5 | 6.63E+12 | 0.19 | 1.92 | 71.90 | 70.50 | 783.30 |
| 22 | 3 | 1 | 3.25 | 17.5 | 4.41E+12 | 0.12 | 2.07 | 75.70 | 69.15 | 807.00 |
| 23**^5^** | 0 | 1 | 3.25 | 17.5 | 5.55E+11 | 0.00 | 3.42 | 98.50 | 1.19 | 127.60 |
| 24 | 7 | 1 | 3.25 | 17.5 | 6.23E+11 | 0.03 | 2.95 | 39.50 | 57.05 | 748.90 |
| 25**^5^** | 3**^7^** | 0 | 3.25 | 17.5 | 5.00E+08 | 0.00 | 1.02 | 98.90 | 1.37 | 0.00 |
| 26 | 3 | 2 | 3.25 | 17.5 | 1.02E+12 | 0.06 | 2.12 | 41.40 | 55.65 | 968.50 |
| 27 | 3 | 1 | 0.5 | 17.5 | 4.49E+12 | 0.11 | 2.56 | 82.30 | 50.70 | 1276.30 |
| 28 | 3 | 1 | 6.75 | 17.5 | 4.87E+12 | 0.11 | 2.63 | 67.80 | 76.95 | 621.80 |
| 29 | 3 | 1 | 3.25 | 0**^8^** | 5.68E+12 | 0.29 | 4.27 | 79.40 | 87.25 | 413.00 |
| 30 | 3 | 1 | 3.25 | 42.5 | 4.06E+12 | 0.08 | 2.54 | 73.70 | 68.15 | 1034.70 |
| 31 | 3 | 1 | 3.25 | 17.5 | 4.08E+12 | 0.12 | 2.21 | 71.40 | 74.00 | 794.20 |
| 32 | 3 | 1 | 3.25 | 17.5 | 4.30E+12 | 0.13 | 2.50 | 71.90 | 77.35 | 779.70 |
| 33 | 3 | 1 | 3.25 | 17.5 | 3.68E+12 | 0.11 | 2.02 | 71.00 | 74.15 | 823.20 |

**^1^**The DoE experiments were conducted in random order, but they are shown in numerical order for summary purposes.

**^2^**Day 3 cell viability is included to represent cumulative cytotoxic effects from initial transfection and rAAV production.. Cell viability was measured by trypan blue staining on the Cedex HiRes (Roche, Switzerland).

**^3^**Average transfection efficiency (%) was calculated by averaging the percentage of enhanced green fluorescent protein positive (eGFP+) cells on days 2 and 3 post-transfection. The percentages of eGFP+ cells were determined by: 1) establishing gates for single cells based on area and aspect ratio, and 2) establishing a gate for eGFP+ cells based on non-transfected HEK293 cells as a negative control on a plot of intensity vs normalized frequency. 10,000 single cells for each flask were analyzed on a Amnis® FlowSight® Imaging Flow Cytometer (MilliporeSigma, USA). A 488 nm laser and a filter with 0.5mW intensity was used to detect eGFP+ cells.

**^4^**PEI& DNA complex size (hydrodynamic diameter in nm) was calculated by averaging technical triplicate measurements obtained by Dynamic Light Scattering (DLS) on a Zetasizer Nano S90 (Malvern Panalytical, UK). The following settings were used for complex size acquisition: 1) Material - PEI, Refractive Index 1.63, 2) Dispersant - water.

**^5^**Run 25 (no PEI, no DNA) and run 23 (no PEI, included DNA) control conditions were excluded from the model and were used to set the qPCR and ELISA LODs.

**^6^**Run 4 was excluded from the model because its qPCR values were at the LOD set by run 25 and run 23.

**^7^**The transfection for run #25 was conducted with 0 pg DNA/cell added to the complexation reaction; therefore, although the MODDE software selected a PEI:DNA of 3, the actual amount of PEI added was zero.”

**^8^**DLS measurements were conducted so that the incubation time was as close to 0 as possible, meaning that polyplex size was measured right after the PEI addition and pipette mixing were completed.

**Table S2:** Primer and probe sequences for polymerase chain reaction (PCR)-based assays

| Target | Primer | | Probe (5’-3’) |
| --- | --- | --- | --- |
|  | Forward (5’-3’) | Reverse (5’-3’) |  |
| eGFP | GAACCGCATCGAGCTGAA | TGCTTGTCGGCCATGATATAG | /56-FAM/ATCGACTTC /ZEN/AAGGAGGACGGCAAC/3IABkFQ/ |
| hBglob polyA tail | gggatttccaagtctccac | caccgtacacgcctacc | /5Cy5/AAATGTCGT/TAO/A ACAACTCCGCCC/3IAbRQSp/ |
| CMV promoter | AACTCCATGAAAGAAGGTGAGG | CTGAGGGATGAATAAGGCATAGG | /5HEX/AGCTAATGC/ZEN/ACATTGGAACAGC/3IABkFQ/ |

**Figure S1:** Capsid particle (CP) titers by enzyme-linked immunosorbent assay (ELISA) for purified reference standards with and without spiked HEK293 lysate used to determine a correlation factor to capture matrix effects.

CP raw titer data for rAAV2 reference standards (Vigene Biosciences, USA) was collected with a rAAV2 ELISA kit and titers were quantified using a standard curve of empty rAAV2 capsids. The reference vectors, AAV2-LacZ, AAV2-GFP, and AAV2-Empty, were diluted in a two-step process. First, each reference vector was diluted in assay buffer or null HEK293 lysate so that its concentration was within range of the DoE samples. Next, the vectors were further diluted using the dilution factors used to run the DoE samples (5x, 25x, and 125x) with assay buffer. The percent decrease in reading for samples diluted in lysate versus those diluted in assay buffer was used to determine the correlation factor of 2 used for DoE sample CP titer correction (Supplemental Table 3). Error bars show standard deviations for technical duplicates, with the exception of the LacZ_125 samples, which were run in quadruplicate.

**Table S3:** Data used to calculate the correlation factor to compensate for matrix effects of HEK293 cell lysate in ELISA assays.

|  | **Dilution 1** (Assay Buffer or Lysate) | **Dilution 2**  (Assay Buffer) | **Total Dilution** | **Conc #1** (CP/L) | **Conc #2** (CP/L) | **Conc #3** (CP/L) | **Conc #4** (CP/L) | **Average Capsid titer**  (CP/L) | **Standard Deviation** | **Reduction Rate** | **Average Reduction Rate** | **Correlation Factor** |
| --- | --- | --- | --- | --- | --- | --- | --- | --- | --- | --- | --- | --- |
| LacZ_25 | 625 | 25 | 15625 | 3.00E+13 | 2.50E+13 |  |  | 2.75E+13 | 3.54E+12 | 61.52% | 53.69% | 2 |
| LacZ_25_lysate | 625 | 25 | 15625 | 1.08E+13 | 1.03E+13 |  |  | 1.06E+13 | 3.78E+11 |  |  |  |
| LacZ_125 | 625 | 125 | 78125 | 2.68E+13 | 2.94E+13 | 2.07E+13 | 2.24E+13 | 2.48E+13 | 4.01E+12 | 49.07% |  |  |
| LacZ_125_lysate | 625 | 125 | 78125 | 1.26E+13 | 1.28E+13 | 1.20E+13 | 1.33E+13 | 1.27E+13 | 5.45E+11 |  |  |  |
| GFP_5 | 25 | 5 | 125 | 2.14E+11 | 2.14E+11 |  |  | 2.14E+11 | 4.30E+08 | 49.67% |  |  |
| GFP_5_lysate | 25 | 5 | 125 | 1.11E+11 | 1.05E+11 |  |  | 1.08E+11 | 4.33E+09 |  |  |  |
| GFP_25 | 25 | 25 | 625 | 2.49E+11 | 2.84E+11 |  |  | 2.66E+11 | 2.51E+10 | 59.73% |  |  |
| GFP_25_lysate | 25 | 25 | 625 | 1.07E+11 | 1.07E+11 |  |  | 1.07E+11 | 2.74E+08 |  |  |  |
| GFP_125 | 25 | 125 | 3125 | 2.93E+11 | 2.92E+11 |  |  | 2.93E+11 | 3.45E+08 | 53.20% |  |  |
| GFP_125_lysate | 25 | 125 | 3125 | 1.43E+11 | 1.31E+11 |  |  | 1.37E+11 | 9.10E+09 |  |  |  |
| Empty_5 | 125 | 5 | 625 | 9.64E+11 | 1.01E+12 |  |  | 9.89E+11 | 3.42E+10 | 49.46% |  |  |
| Empty_5_lysate | 125 | 5 | 625 | 4.65E+11 | 5.35E+11 |  |  | 5.00E+11 | 4.93E+10 |  |  |  |
| Empty_25 | 125 | 25 | 3125 | 1.07E+12 | 1.06E+12 |  |  | 1.07E+12 | 9.48E+09 | 53.18% |  |  |
| Empty_25_lysate | 125 | 25 | 3125 | 5.02E+11 | 4.98E+11 |  |  | 5.00E+11 | 3.40E+09 |  |  |  |
| Empty_125 | 125 | 125 | 15625 | 1.16E+12 | 1.09E+12 |  |  | 1.12E+12 | 4.94E+10 | 55.87% |  |  |
| Empty_125_lysate | 125 | 125 | 15625 | 4.78E+11 | 5.13E+11 |  |  | 4.96E+11 | 2.46E+10 |  |  |  |

Capsid particle (CP) titers were measured using the PROGEN ELISA 2.0R kit, and the data obtained from samples with and without HEK293 cell lysates were directly compared. On average, there was a 53.96% reduction in capsid titer in the samples that were diluted with HEK293 cell lysates versus those diluted only in assay buffer. This indicates the presence of HEK293 cell lysate in a sample causes suppressive matrix effects that reduces the final capsid titer value by approximately 50%, as observed for all three reference vectors across a wide range of dilutions (125~78125x). These data indicate that capsid titers should be multiplied by the correlation factor 2 when using the ELISA kit and the capsid concentration prevents sufficient dilution of the lysate.

**Figure S2:** Vector genome (VG) titers targeting eGFP for the 33 DoE conditions.

VG titer raw data was collected using quantitative PCR (qPCR) and titers were quantified with a diluted rAAV5-eGFP vector standard curve. Error bars show standard deviations for technical duplicates.

**Figure S3**: Capsid particle (CP) titer for the 33 DoE conditions with technical duplicates.

CP titer raw data was collected using an AAV2 ELISA and titers were quantified using a standard curve of empty rAAV2 capsids. Error bars show standard deviations for technical duplicates.


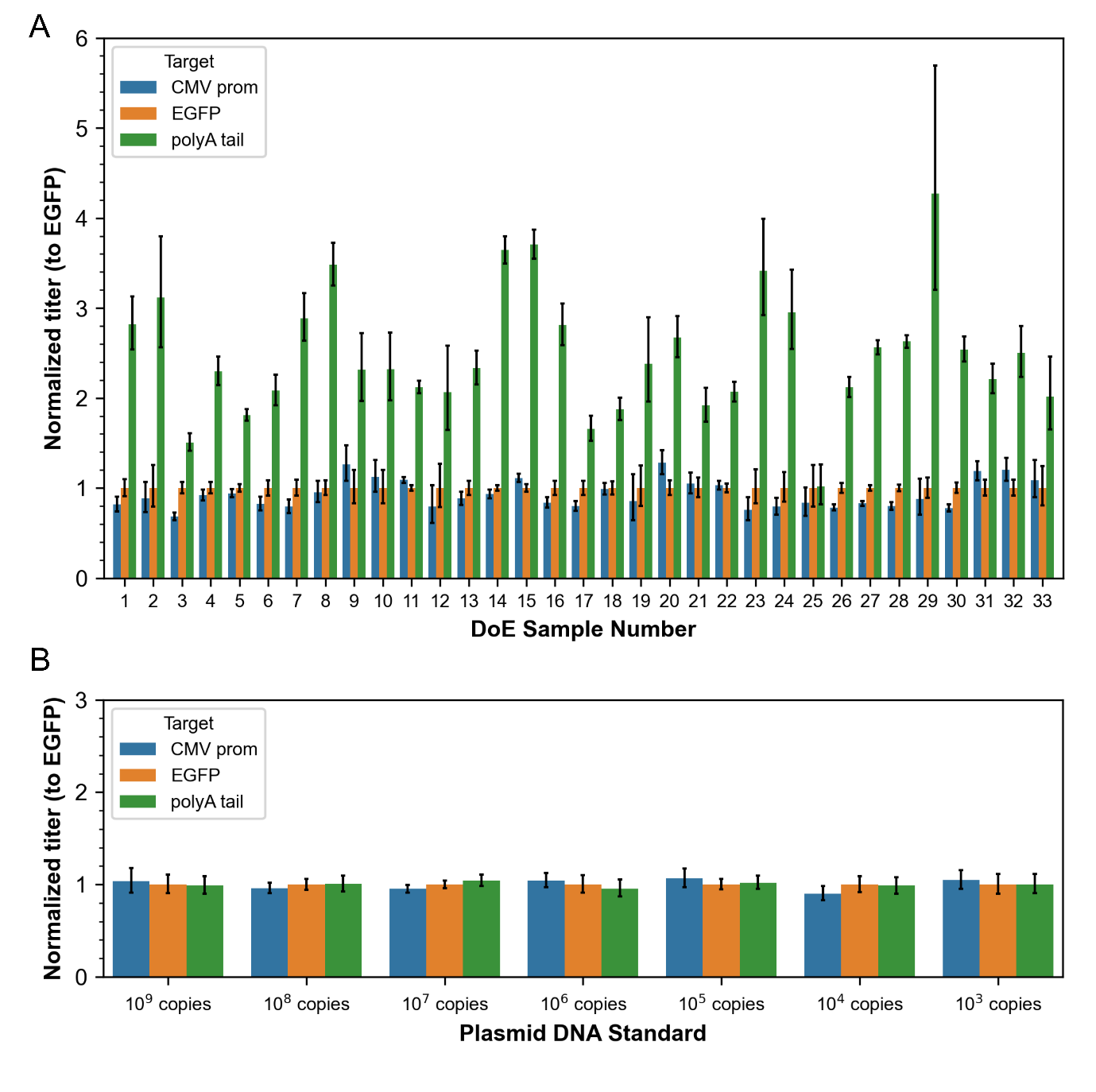


**Figure S4:** Three-plex VG titer data normalized to the eGFP transgene.

Multiplex VG titer raw data was collected using quantitative PCR (qPCR) and titers were quantified for each target using a diluted pAAV-CMV-EGFP (pGoI) standard curve. Calculated VG titers for each target were then normalized to the eGFP titer for comparative analysis. Samples with a normalized titer value of ~1 for all targets likely contain a higher percentage of full genomes than samples with any normalized titers that are significantly higher or lower than 1. The normalized polyA VG titer values were used as the 2D VG titer output for the model because they showed the most variability between DoE conditions, with average values ranging from ~1-4. Statistical analysis using Student’s t-test showed that p < 0.05 when comparing the normalized polyA tail titers to the normalized eGFP titers for all DoE and verification conditions except the no PEI control (DoE sample 25) and plasmid standards. Error bars show standard deviations for technical quadruplicates.

**Table S4:** Regression coefficients (coef) and associated probability values (p-value) for PQAs used for model fitting.

| Model Terms | Y_1_ |  | Y_2_ |  | Y_3_ |  |
| --- | --- | --- | --- | --- | --- | --- |
|  | Coef. | p-value | Coef. | p-value | Coef. | p-value |
| Constant | 12.69 | 0.00E+00* | 0.19 | 7.42E-07* | 0.37 | 1.25E-14* |
| X1 | -0.29 | 2.61E-10* | -0.03 | 2.34E-01 | 0.03 | 7.01E-02 |
| X2 | -0.26 | 2.74E-9* | -0.12 | 2.24E-05* | 0.01 | 4.16E-01 |
| X3 | -0.03 | 3.17E-01 | 0.04 | 9.67E-02 | -- | -- |
| X4 | 0.03 | 2.25E-01 | -0.05 | 4.97E-02* | -0.03 | 2.96E-02* |
| X1×X2 | -0.15 | 3.80E-06* | -- | -- | -0.05 | 5.89E-04* |
| X1×X3 | -- | -- | -- | -- | -- | -- |
| X1×X4 | -- | -- | -0.05 | 6.41E-03* | -- | -- |
| X2×X3 | -- | -- | -0.05 | 5.83E-02 | -- | -- |
| X2×X4 | -- | -- | -- | -- | -- | -- |
| X3×X4 | 0.10 | 1.69E-03* | -- | -- | -- | -- |
| X1×X1 | -- | -- | -- | -- | -- | -- |
| X2×X2 | -- | -- | 0.06 | 7.25E-03* | -0.01 | 2.93E-01 |
| X3×X3 | -- | -- | -- | -- | -- | -- |
| X4×X4 | -- | -- | -- | -- | 0.03 | 1.62E-02* |

“*” Significant term with p-value < 0.05; “--” The term has no or a negative impact for model fit.


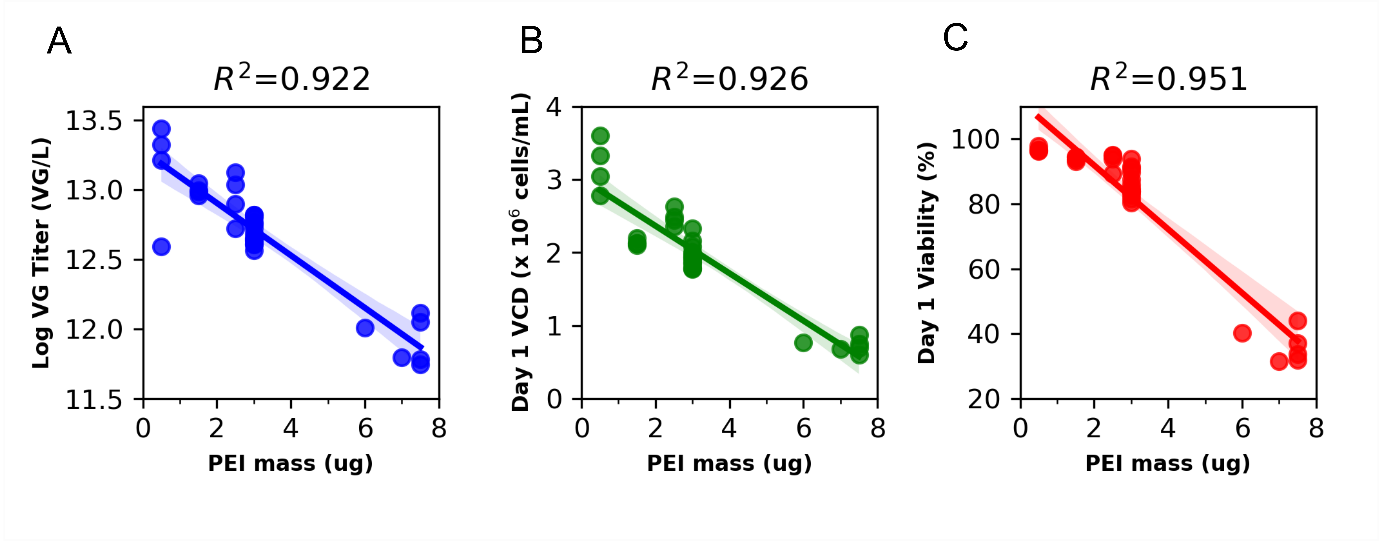


**Figure S5:** Regression plots showing first order relationships between input PEI mass and process variable or quality attribute outputs

PEI mass had linear relationships with **(S5A)** the base 10 logarithm (Log) of vector genome (VG) titer measured from cell culture harvested day 3 post-transfection **(S5B)** viable cell density (VCD) day 1 post-transfection, and **(S5C)** cell viability day 1 post-transfection. The goodness of fit, R^2^, is show above each plot, and the colored banding indicates the 95 percent confidence interval.


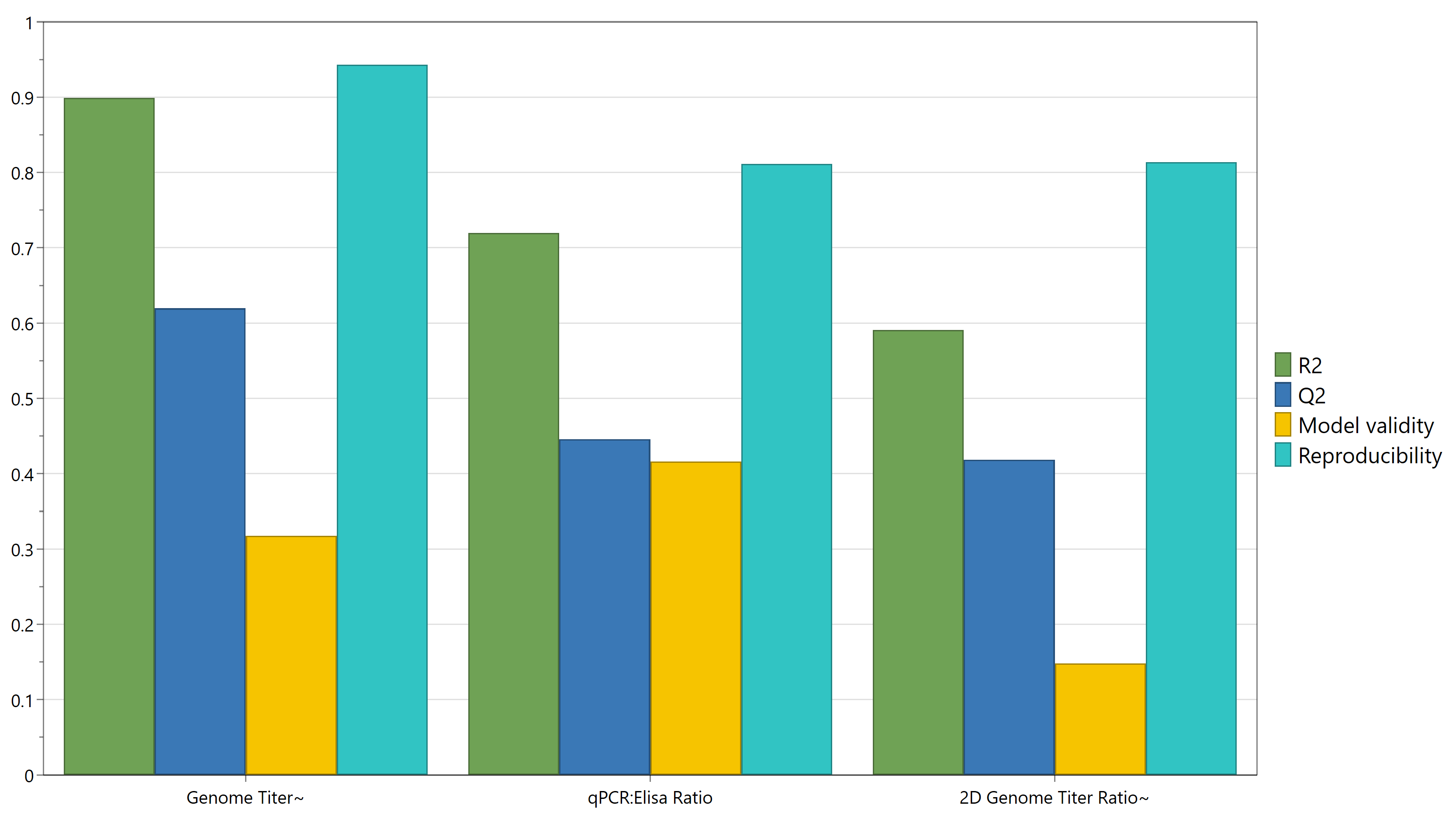


**Figure S6:** Parameters used to evaluate PQA model fits

The coefficient of determination (R2), coefficient of prediction (Q2), model validity, and reproducibility for the final PQA models. PQA data sets that underwent logarithmic transformation are marked with a tilde (~) on the x-axis. Note: Genome Titer = VG Titer, qPCR:ELISA ratio = VG:CP ratio, 2D Genome Titer Ratio = 2D VG Titer Ratio.


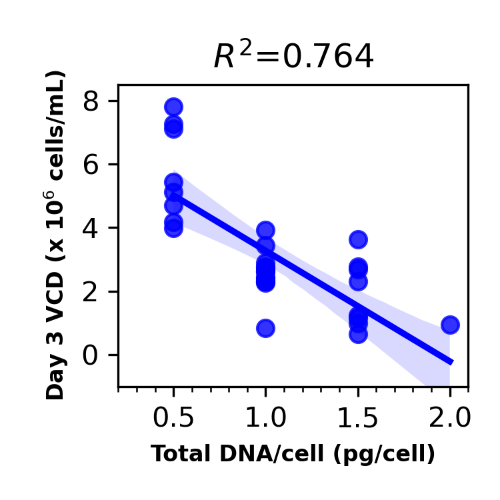


**Figure S7:** Regression plot showing first order relationship between input DNA mass per cell and VCD

The total DNA mass per cell added to the complexation reaction had a linear relationship with viable cell density (VCD) day 3 post-transfection (harvest day). The goodness of fit, R^2^, is show above the plot, and the colored banding indicates the 95 percent confidence interval.

**
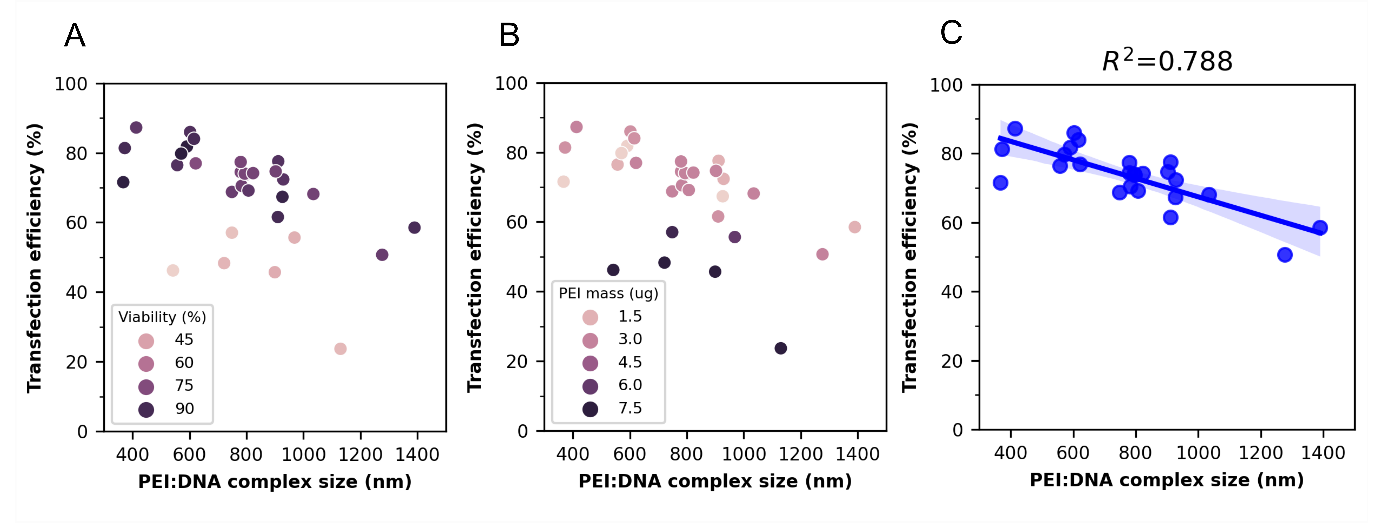
**

**Figure S8:** Scatter and regression plots showing first order relationship between transfection efficiency and PEI/DNA complex size

Scatter plots depicting the linear relationship between transfection efficiency and PEI/DNA complex size are shown shaded by **(S8A)** cell viability and **(S8B)** total PEI mass. Cultures with cell viability > 50% have a high R^2^ correlation between transfection efficiency and PEI/DNA complex size as shown by regression plotting, with colored banding indicating the 95 percent confidence interval **(S8C)**.

**Abbreviations:**

AAV, adeno-associated virus; CCO, central composite orthogonal; CMV, cytomegalovirus; CP, capsid particle; CQA, critical quality attribute; DoE, design of experiments; eGFP, enhanced green fluorescent protein; ELISA, enzyme-linked immunosorbent assay; hBglob, human β-globin; HEK293, human embryonic kidney 293; PP, process parameter; PCR, polymerase chain reaction; PEI, polyethyleneimine; pg, picograms; polyA tail, polyadenylation tail; prom, promoter; qPCR, quantitative polymerase chain reaction; rAAV, recombinant adeno-associated virus; VCD, viable cell density; VG, vector genome;
